# Identification of new susceptibility loci for IgA nephropathy in Han Chinese

**DOI:** 10.1038/ncomms8270

**Published:** 2015-06-01

**Authors:** Ming Li, Jia-Nee Foo, Jin-Quan Wang, Hui-Qi Low, Xue-Qing Tang, Kai-Yee Toh, Pei-Ran Yin, Chiea-Chuen Khor, Yu-Fen Goh, Ishak D. Irwan, Ri-Cong Xu, Anand K. Andiappan, Jin-Xin Bei, Olaf Rotzschke, Meng-Hua Chen, Ching-Yu Cheng, Liang-Dan Sun, Geng-Ru Jiang, Tien-Yin Wong, Hong-Li Lin, Tin Aung, Yun-Hua Liao, Seang-Mei Saw, Kun Ye, Richard P. Ebstein, Qin-Kai Chen, Wei Shi, Soo-Hong Chew, Jian Chen, Fu-Ren Zhang, Sheng-Ping Li, Gang Xu, E. Shyong Tai, Li Wang, Nan Chen, Xue-Jun Zhang, Yi-Xin Zeng, Hong Zhang, Zhi-Hong Liu, Xue-Qing Yu, Jian-Jun Liu

**Affiliations:** 1 Department of Nephrology, The First Affiliated Hospital, Sun Yat-sen University, Guangzhou , Guangdong 510080, China; 2 Key Laboratory of Nephrology, Ministry of Health and Guangdong Province, Guangzhou, Guangdong 510080, China; 3 Human Genetics, Genome Institute of Singapore, Agency for Science, Technology and Research, Singapore 138672, Singapore; 4 National Clinical Research Center of Kidney Diseases, Jinling Hospital, Nanjing University School of Medicine, Nanjing, Jiangsu 210002, China; 5 Singapore Eye Research Institute, Singapore 169856, Singapore; 6 Department of Ophthalmology, Yong Loo Lin School of Medicine, National University of Singapore and National University Health System, Singapore 119228, Singapore; 7 Singapore Immunology Network, Agency for Science, Technology and Research, Singapore 138648, Singapore; 8 Department of Nephrology, General Hospital of Ningxia Medical University, Yinchuan, Ningxia 750004, China; 9 Institute of Dermatology and Department of Dermatology, No.1 Hospital, Anhui Medical University, Hefei, Anhui, 230032, China; 10 State Key Laboratory Incubation Base of Dermatology, Ministry of National Science and Technology, Hefei, Anhui 230032, China; 11 Department of Nephrology, XinHua Hospital, School of Medicine, Shanghai Jiao Tong University, Shanghai 200092, China; 12 Department of Nephrology, The First Affiliated Hospital, Dalian Medical University, Dalian, Liaoning 116011, China; 13 Department of Nephrology, The First Affiliated Hospital, Guangxi Medical University, Nanning 530021, China; 14 Saw Swee Hock School of Public Health, National University of Singapore, National University Health System, Singapore 117549, Singapore; 15 Department of Nephrology, The People's Hospital of Guangxi Autonomous Region, Nanning, Guangxi 530021, China; 16 Department of Psychology, National University of Singapore, Singapore 117570, Singapore; 17 Department of Nephrology, the First Affiliated Hospital of Nanchang University, Nanchang, Jiangxi 330006, China; 18 Department of Nephrology, Guangdong General Hospital, Guangzhou, Guangdong 510080, China; 19 Department of Economics, National University of Singapore, Singapore 117570, Singapore; 20 Department of Nephrology, Fuzhou General Hospital of Nanjing Military Command, Fuzhou, Fujian 350025, China; 21 Shandong Provincial Institute of Dermatology and Venereology, Shandong Academy of Medical Science, Jinan, Shandong 250000, China; 22 Department of Hepatobiliary Oncology, State Key Laboratory of Oncology in South China, Sun Yat-sen University Cancer Center, Guangzhou, Guangdong 510080, China; 23 Department of Nephrology, Tongji Hospital, Tongji Medical College of Huazhong University of science & Technology, Wuhan, Hubei 430030, China; 24 Duke-National University of Singapore Graduate Medical School, Singapore 169857, Singapore; 25 Department of Medicine, Yong Loo Lin School of Medicine, National University of Singapore, National University Health System, Singapore 119228, Singapore; 26 Department of Nephrology, Sichuan Provincial People's Hospital, Chengdu, Sichuan 610072, China; 27 Department of Nephrology, RuiJin Hospital, School of Medicine, Shanghai Jiao Tong University, Shanghai 200025, China; 28 State Key Laboratory of oncology in South China, Sun Yat-sen University Cancer Center, Guangzhou 510080, China; 29 Peking Union Medical College, Chinese Academy of Medical Science, Beijing 100730, China; 30 Renal Division, Peking University First Hospital, Peking University, Institute of Nephrology, Beijing 100034, China; 31 School of Biological Sciences, Anhui Medical University, Hefei, Anhui 230032, China; 32 Institute of Dermatology and Department of Dermatology, No.1 Hospital, Anhui Medical University, Hefei, Anhui 230032, China

## Abstract

IgA nephropathy (IgAN) is one of the most common primary glomerulonephritis. Previously identified genome-wide association study (GWAS) loci explain only a fraction of disease risk. To identify novel susceptibility loci in Han Chinese, we conduct a four-stage GWAS comprising 8,313 cases and 19,680 controls. Here, we show novel associations at *ST6GAL1* on 3q27.3 (rs7634389, odds ratio (OR)=1.13, *P*=7.27 × 10^−10^), *ACCS* on 11p11.2 (rs2074038, OR=1.14, *P*=3.93 × 10^−9^) and *ODF1-KLF10* on 8q22.3 (rs2033562, OR=1.13, *P*=1.41 × 10^−9^), validate a recently reported association at *ITGAX-ITGAM* on 16p11.2 (rs7190997, OR=1.22, *P*=2.26 × 10^−19^), and identify three independent signals within the *DEFA* locus (rs2738058, *P*=1.15 × 10^−19^; rs12716641, *P*=9.53 × 10^−9^; rs9314614, *P*=4.25 × 10^−9^, multivariate association). The risk variants on 3q27.3 and 11p11.2 show strong association with mRNA expression levels in blood cells while allele frequencies of the risk variants within *ST6GAL1*, *ACCS* and *DEFA* correlate with geographical variation in IgAN prevalence. Our findings expand our understanding on IgAN genetic susceptibility and provide novel biological insights into molecular mechanisms underlying IgAN.

## Abstract (web summary)

IgA nephropathy is a major cause of end-stage renal disease in China, occurring at a high frequency in Asian populations. Here Li and colleagues conduct a four-stage genome-wide association study in a Chinese population, identifying novel loci and variants associated with disease risk.

## Graphical abstract

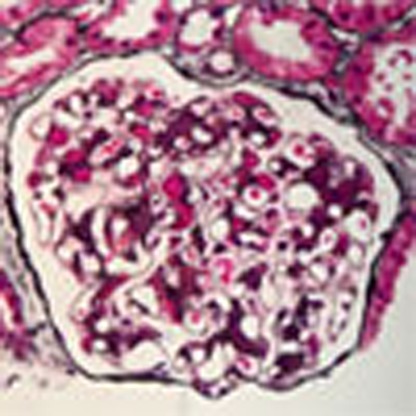

IgA nephropathy (IgAN) is the most common primary glomerulonephritis and a major cause of end-stage renal disease in the Chinese population. It is characterized by the deposition of IgA in the mesangial area of glomeruli, and up to 40% of the cases progress to end-stage renal diseases within 20 years of disease onset^1^,^2^. There is a marked regional difference in frequency of IgAN. It occurs with highest frequency in Asian populations, accounting for 45–58.2% of primary glomerular disease, with modest frequency in Caucasians (USA and Europe) and with lower frequency in the African population^2^,^3^. These differences, together with evidence of familial clustering, strongly suggest the presence of a substantial genetic contribution to the disease. Previous genome-wide association studies (GWASs) have identified five loci significantly associated with IgAN, namely chromosome 1q32 (*CFHR3*-*CFHR1* genes), 6p21 (MHC), 8p23 (*DEFA* gene cluster), 17p13.1 (*TNFSF13*) and 22q12 (*HORMAD2*), providing the first valuable insights into genetic risk factors underlying disease mechanisms^4^,^5^. However, these explain only a fraction of the disease risk and it is clear that more genes and loci remain to be discovered.

In this study, we conduct a four-stage GWAS comprising 8,313 cases and 19,680 controls of Han Chinese ancestry (Supplementary Fig. 1). We identify novel loci on 3q27.3 (*ST6GAL1*), 11p11.2 (*ACCS*) and 8q22.3 (*ODF1-KLF10*), validate a recently reported association on 16p11.2 (*ITGAX-ITGAM*), and identify three independent signals within the *DEFA* locus on 8p23. These findings significantly expand our understanding of the genetic susceptibility to IgAN.

## Results

### Genome-wide discovery analysis

We performed a new GWAS analysis (stage 1) by combining our published GWAS data set^5^ (consisting 1,434 cases and 4,270 controls) with an additional 6,511 control subjects of Chinese Han ethnicity, including 981 subjects from Guangdong, 523 subjects from Shandong and 5,007 subjects from Singapore (Supplementary Table 1). In each data set, we imputed untyped single-nucleotide polymorphisms (SNPs) using the 1,000-genome multi-ethnic reference panel (Feb 2012, IMPUTE v2) (refs 6, 7, 8). After merging shared SNPs across the data sets and stringent quality control filtering (see Methods), we analysed a total of 3,792,949 autosomal SNPs in 1,434 cases and 10,661 controls. With the expanded sample size and deep imputation of untyped variants, this new GWAS analysis has improved statistical power over the previous GWAS to detect risk variants in the range of odds ratio (OR) <1.3 and minor allele frequency (MAF) <10% (Supplementary Table 2)^9^. We analysed genotype dosages taking into account imputation uncertainties^10^, using a logistic regression model assuming an additive effect for allelic risk and adjusting for population stratification using the first five principal components (PCs) as covariates (Supplementary Figs 2–5). The genomic inflation factor after PC correction was low (*λ*_GC_=1.087; 1.073 for SNPs with MAF ≥5%, *λ*_1,000_=1.034), suggesting minimal effects of population stratification in our discovery GWAS. In addition, we examined genotypes of 3,731,832 SNPs imputed with high confidence (info score >0.8, with call rates >95% for genotype with probabilities >0.9) using a multivariate linear mixed model implemented in GEMMA^11^,^12^ (*λ*_GC_=1.039; 1.015 for SNPs with MAF ≥5%, *λ*_1,000_=1.015) (Supplementary Figs 2 and 3), which gave results that were largely consistent with the logistic regression analysis with PC correction (Tables 1 and 2, Supplementary Data 1).

Our genome-wide discovery analysis provided strong supporting evidence for previously published associations^4^,^5^ at 17p13 and 22q12 (Supplementary Table 3, Supplementary Figs 6–8). A similar trend of association was also observed at 1q32, even though the result was not statistically significant (Supplementary Table 3). We also observed strong and consistent evidence for the three independent association signals within the major histocompatibility complex (MHC) region 6p21 that we previously reported (Supplementary Table 3). In addition, we identified a novel independent signal at rs2295119 in the MHC region that remained genome-wide significant after conditioning on all previously reported human leukocyte antigen (HLA) SNPs (OR=1.359, logistic regression *P*_unconditioned_=3.24 × 10^−11^, *P*_conditioned_=7.52 × 10^−10^). This SNP is in linkage disequilibrium (LD) with rs9277554 (*r*^2^=0.556, *D*′=0.993) which was found to be independently associated but did not reach genome-wide significance in our previous study^5^.

We performed HLA imputation on the expanded GWAS data set using genotyped SNPs within chr6: 20–40 Mb (build 37), the SNP2HLA tool^13^ and the Pan-Asian reference panel^14^,^15^. In addition to the associations at the previously reported four-digit HLA alleles (Supplementary Table 4), we also observed strong associations at two-digit alleles DPB1*02 (OR=1.32, logistic regression *P*=1.77 × 10^−9^) tagged by rs2295119 (*r*^2^=0.94), and DRB1*04 (OR=1.45, logistic regression *P*=3.16 × 10^−11^), tagged by previously reported SNPs rs660895 (*r*^2^=0.44) and rs1794275 (*r*^2^=0.13) (Supplementary Table 4). Detailed HLA sequencing and typing analyses will be needed to further understand these associations.

Our analysis also suggested three independent association signals within the *DEFA* gene cluster on 8p23 (each *P*<10^−4^, *r*^2^<0.1 between each pair of SNPs). After excluding the SNPs within the five previously identified regions, a notable excess of extremely small *P* values was observed on the quantile–quantile plot compared with the expected null distribution (Supplementary Fig. 9), which suggests the existence of additional associations beyond the ones already identified.

### Validation analysis

We first selected the top 136 independent SNPs exceeding *P* <1 × 10^−4^ in either the PC-adjusted logistic regression or GEMMA analysis that are not within the known loci (hypothesis free). Finally, by including the three independent SNPs within the 8p23 locus, a total of 139 SNPs were selected for the first validation study, but only the assays for 122 were successfully designed for multiplex genotyping analysis by Sequenom.

As the initial validation, 115 SNPs were successfully genotyped in 2,651 IgAN cases and 2,907 controls of Han Chinese ethnicity recruited from the Southern region of China (stage 2). We performed logistic regression analysis of the validation samples and combined the association statistics across the discovery (PC-adjusted or GEMMA) and validation samples by a fixed-effects meta-analysis (Supplementary Data 1). We then took forward the top 22 SNPs with *P*<1 × 10^−4^ in the meta-analysis of the combined stages 1 and 2 samples for further validation analysis in an independent set of samples (stage 3) consisting of 2,428 IgAN cases and 4,202 controls recruited from the Northern (1,463 cases and 1,683 controls) and Southern (965 cases and 2,519 controls) regions of China (Supplementary Table 5). Finally, the top eight SNPs with *P*<5 × 10^−7^ in the combined samples of stages 1+2+3 and showing consistent associations across all four sample collections were analysed in an additional independent set of 1,800 IgAN cases and 1,910 controls (stage 4) recruited from Northern (704 cases and 805 controls) and Southern (1,096 cases and 1,105 controls) China (Table 1). We then conducted a full meta-analysis of all the stages 1–4 samples (Supplementary Fig. 1) by analysing the Northern and Southern samples of each of the four stages as independent sample collections (six independent samples in total) to minimize bias resulting from population stratification.

**Figure 1:**
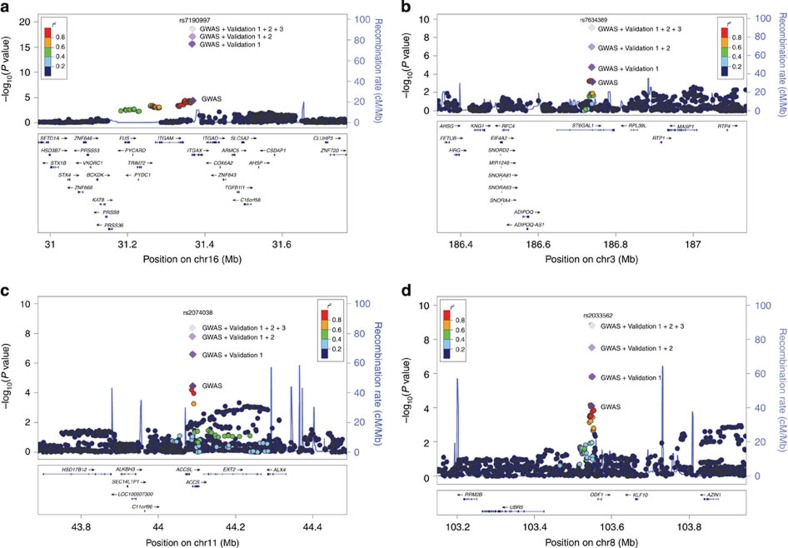
Recombination plots of the novel loci reaching genome-wide significance. (**a**) rs7190997 at 16p11.2, (**b**) rs7634389 at 3q27.3, (**c**) rs2074038 at 11p11.2 and (**d**) rs2033562 at 8q22.3, showing *P* values obtained in the GWAS discovery (logistic regression) and in the combined analysis of GWAS and validation 1, 2 and 3 samples (fixed-effects meta-analysis).

**Figure 2:**
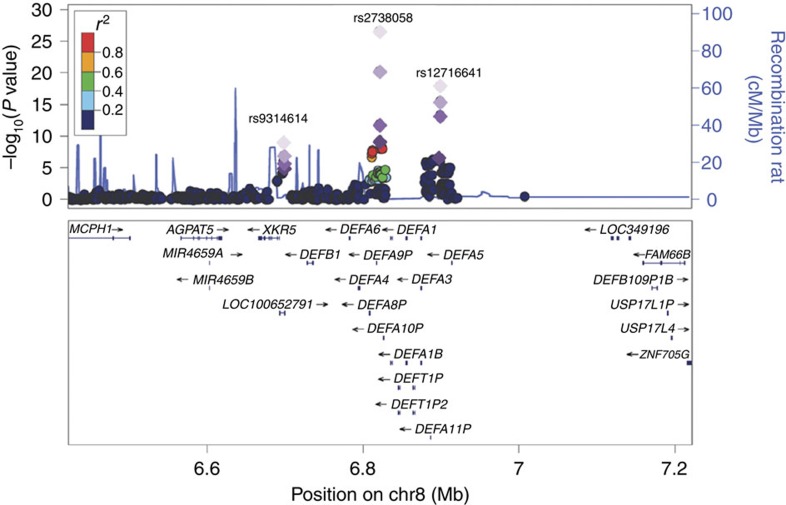
Recombination plots of the three independent loci at the defensin locus, showing *P* values obtained in the GWAS discovery (logistic regression) and in the combined analysis of GWAS and validation 1, 2 and 3 samples (fixed-effects meta-analysis). The two novel signals are in low linkage disequilibrium with rs2738058, which tags the previously reported SNP rs2738048 and are separated from these SNPs by regions of high recombination rates.

From the combined analysis of a total of 8,313 IgAN cases and 19,680 controls, we identified SNPs at four out of the five novel loci reaching genome-wide significance (*P*<5 × 10^−8^), rs7190997 within the *ITGAM-ITGAX* locus on 16p11.2 (OR=1.22, fixed-effects meta-analysis *P*=2.26 × 10^−19^), rs2074038 at the *ACCS* locus on 11p11.2 (OR=1.14, meta-analysis *P*=3.93 × 10^−9^), rs2033562 near *ODF1-KLF10* on 8q22.3 (OR=1.13, meta-analysis *P*=1.41 × 10^−9^) and rs7634389 at the *ST6GAL1* locus on 3q27.3 (OR=1.13, meta-analysis *P*=7.27 × 10^−10^) (Table 1, Fig. 1). The fifth locus rs11264799 (*FCRL3)* on 1q23.1 remained suggestive (OR=1.14, meta-analysis *P*=2.00 × 10^−7^) (Supplementary Table 6). We also confirmed all three independent association signals within the *DEFA* locus at rs2738058 (OR=1.23, meta-analysis *P*=1.15 × 10^−19^), rs12716641 (OR=1.15, meta-analysis *P*=9.53 × 10^−9^) and rs9314614 (OR=1.13, meta-analysis *P*=4.25 × 10^−9^) through a multivariate association analysis (Table 2, Fig. 2). All the novel associations showed consistent effects across all the independent sample collections without evidence of heterogeneity and obtained statistically significant in the validation samples after correction for multiple testing (Bonferroni corrected *P*<0.05/154=3.25 × 10^−4^; Tables 1 and 2).

To further ensure that none of the novel associations were influenced by population stratification and/or batch effects among the expanded control samples of stage 1 (genotyped in different arrays), we re-examined the imputation info scores and allele frequencies of all the validated SNPs across the different control data sets and found them to be high quality and very consistent across genotyping arrays and sample collections (Supplementary Table 7). We re-examined our reported SNPs in a subset of samples (1,434 cases and 4,270 controls) that were analysed in our previous GWAS^5^, on which the imputation was performed as a batch from 444,882 overlapping autosomal SNPs that were genotyped on different Illumina chips and found that the strengths of these associations (ORs) were consistent with the results from the full data set, indicating that the new evidences of these associations were driven by improved statistical power and genetic variation coverage rather than systematic bias due to the inclusion of additional control samples that were imputed in separate data sets (Supplementary Table 7). Good genotyping clusters were observed across all genotyping platforms for the reported SNPs (Supplementary Figs 10–12) with allele frequencies of imputed SNPs closely matching those genotyped in the validation samples (Tables 1 and 2).

Next, we re-ran the full meta-analysis by dividing the discovery data set into Northern (414 cases and 2,306 controls) and Southern (1,020 cases and 8,355 controls) clusters with adjustment of the top five principal components re-calculated within each cluster (see Methods) (Supplementary Fig. 5), and the full meta-analysis results were consistent at the top loci (Supplementary Table 8). The association effects were not significantly different between the combined (discovery and validation) Northern and Southern samples (*P*_het_>0.05) (Supplementary Table 9). In addition, we did another full meta-analysis with lambda GC correction of the PC-adjusted results from the discovery data set. All the novel loci remained genome-wide significant (Supplementary Table 9). Finally, the associations were not influenced by age and gender, and similar effects were observed in males and females (Supplementary Table 10).

While this manuscript was under preparation, an independent GWAS on IgAN conducted in Europeans and Asians was published and reported three novel loci *ITGAX*, *VAV3* and *CARD9* (ref. 16). Of the two previously reported independent associations within *ITGAX* locus, our top SNP (rs7190997) at *ITGAX* shows a high LD with the reported SNP rs11150612 (*r*^2^=0.877, *D*′=0.988). The other reported SNP rs11574637 as well as SNPs in LD with it are either very rare (MAF<1%) or absent in both our samples and HapMap Asians (http://hapmap.ncbi.nlm.nih.gov/). At the other two novel loci *VAV3* and *CARD9*, we observed a similar direction of association at the reported SNPs^16^ but the results did not reach statistical significance in our discovery samples (*P*>0.05; Supplementary Table 3, Supplementary Fig. 8).

We have also done further investigation of the reported association at the *CFH* locus^4^,^16^ by genotyping rs6677604 in our validation 2 and 3 samples and analysing the Northern and Southern samples of each collection separately in the meta-analysis of the combined discovery and validation samples (Supplementary Table 11). We observed significant association of the *CFH* locus (OR=1.19 (95% confidence interval (CI)=1.07–1.31), meta-analysis *P*=0.0011) without evidence of heterogeneity across all the six sample collections (*I*^2^=0%, Cochrane's *Q* test *P*_heterogeneity_=0.70). The effect size in our samples was, however, smaller than what was previously reported, and a slightly larger effect was observed in our Northern (OR=1.26) than Southern (OR=1.12) samples (Supplementary Table 11).

### Independent associations within the *DEFA* locus

We discovered three independent signals at the *DEFA* locus (Table 2, Supplementary Table 12). Of these, only rs2738058 is in LD with the previously reported SNP rs2738048 (*r*^2^=0.71) (ref. 5), and the other two signals at rs12716641 and rs9314614 were located 76–77 kb and 124–125 kb away from rs2738058 and rs2738048, respectively (Fig. 2). While rs12716641 is located within the *DEFA* gene cluster, rs9314614 is located in the intron of the long-coding RNA *GS1-24F4.2* and separated from the *DEFA* gene cluster by two recombination hotspots, likely representing an independent novel locus. Furthermore, all three are poorly correlated with rs10086568, an independent association within the *DEFA* locus reported in ref. 16 (*r*^2^<0.1 in our samples and 1,000 genomes Asians, although *r*^2^=0.17 with rs12716641 in Europeans). Multiple copy-number variants (CNVs) of *DEFA1-A3* exist within this region^17^ and were previously found to be associated with Crohn's disease^18^ and severe sepsis^19^. Recently, rs4300027 has been reported to tag the CNVs in Europeans (*r*^2^=0.35) (ref. 20). rs4300027 is in moderate LD with rs2738048 (*r*^2^=0.15), rs2738058 (*r*^2^=0.12) and rs12716641 (*r*^2^=0.28), but not rs9314614 (*r*^2^=0.002). This suggests that the associations at rs2738048/rs2738058 and rs12716641 may implicate the role of *DEFA1-A3* CNVs in IgAN. Further fine mapping and functional study will be needed to investigate the association of the *DEFA1-A3* CNVs with IgAN and its relationship with the complex association patterns at these SNPs in Europeans and Asians.

### Functional investigation through eQTL (expression quantitative trait loci) and GRAIL (genetic relationships across implicated loci) analyses

We then investigated potential biological effects of the novel associated SNPs and loci by looking for effects on mRNA expression levels (eQTLs)^21^,^22^, ENCODE annotations of associated variants^23^,^24^, documented associations with other diseases and known biological functions of nearby genes (Table 3, Supplementary Tables 13–15, Supplementary Data 2 and 3). Three out of the four novel loci showed strong associations with the expression levels of the genes *ITGAX, ITGAM*, *ACCS, EXT2* and *ST6GAL1* in peripheral blood cells and others, suggesting an important role of regulatory variants of these genes in IgAN risk. All five SNPs also tag variants that lie in predicted protein binding sites. Although no strong eQTL effects were observed for rs2033562 near *ODF1-KLF10* in blood cells, ENCODE annotations suggest it may play a role in the expression of the nearby *UBR5* gene^24^. Furthermore, although there was no evidence for eQTL effects of rs2738048/rs2738058 and rs12716641, a moderate association with expression of the gene *DEFB1* was observed at rs9314614 in monocytes (linear model *P*=4.22 × 10^−4^; Supplementary Tables 13 and 15)^22^, providing further support that this SNP could tag a novel independent locus and pathway despite its close proximity to the *DEFA* cluster.

These variants are also not in strong LD with those previously associated with other traits and diseases in Europeans or Asians (Table 3, Supplementary Table 13)^25^, and a GRAIL (https://www.broadinstitute.org/mpg/grail/)^26^ analysis of previously reported and novel loci did not identify any major pathways that can account for their associations with IgAN. We anticipate that these loci highlight multiple different pathways including mucosal immune response^16^ that may jointly influence IgAN pathogenesis through changes in gene expression levels. Since most of the existing expression data sets were generated in healthy individuals that were mostly of European descent^21^,^22^, a more careful analysis will be needed to examine the association of these genotypes on the expression levels of these genes in Asian subjects, and to evaluate their biological and clinical relevance in IgAN patients and controls.

At the *ST6GAL1* locus, we observed a trend of increasing allele frequencies of risk allele C at rs7634389 from Africans (16%), Europeans (37.5%) to Asians (40%) in HapMap populations (Supplementary Table 16). Similarly, the *ACCS* SNP rs2074038 risk allele T is absent in Africans, present at moderate frequency in Europe (12%) and highest frequency in Asia (33%). Similar observations were made at two independent variants in the *DEFA* cluster (Supplementary Table 16). Consistent with previous reports on the geographical distribution of risk variants within 1q32, 22q12 (ref. 1), and more recently, 16p11.2 (ref. 16), these trends of increasing risk allele frequencies suggest that pooled differences in risk allele frequencies of these loci combined may contribute to differences in disease prevalence across different world populations^1^,^4^,^16^.

## Discussion

This study has several advantages over our previous study. Firstly, we have performed deep genome-wide imputation in the discovery samples, leading to the discovery of loci tagged by SNPs that were not previously represented on SNP arrays. Secondly, the addition of 6,391 controls led to a moderate gain in statistical power to detect risk alleles with small effect sizes (OR<1.2). Thirdly, we have expanded our validation data sets to include both Northern and Southern Chinese samples that have provided firm independent replication of the association signals. We used geographic matching as a proxy for genetic matching to control the effects of population stratification in our validation data sets, as has been done in previous studies on the Chinese population^27^, although ancestry informative markers may also be helpful. We also acknowledge a number of limitations. Despite deep imputation, we expect that the coverage of low frequency and rare variants (minor allele frequencies <5%) is limited given that most of the imputed rare variants in our study had a low information score that did not pass our strict quality control thresholds and were therefore excluded from further analysis. Also, with the current sample size, we had limited power to analyse lower-frequency variants with minor allele frequencies below 5% (Supplementary Table 2)^9^. Further studies will be needed to directly sequence or genotype rare variants on a larger number of cases and controls to evaluate their role in IgAN risk.

We have conducted the largest study on IgAN in the Han Chinese population to date by analysing a total of 8,313 IgAN cases and 19,680 controls. We have discovered three new loci at 11p11.2, 8q22.3 and 3q27.3, two novel independent associations within the *DEFA-DEFB* gene cluster and validated the recently reported locus at 16p11.2 (ref. 16). We estimate that these novel association signals explain about 1.7% of the disease variance and 5.5% of the variance in combination with the previously published loci^4^,^5^. Some risk variants show significant difference in frequency and may contribute to IgAN prevalence differences across world populations. Our study has significantly expanded our understanding on the genetic basis of IgAN susceptibility and provided novel insight into the mechanisms underlying the development of IgAN.

## Methods

### Study subjects

The original genome-wide discovery analysis involved 1,523 cases (from Southern China) and 4,276 controls (972 controls from southern China, 1,228 controls from Northern China and 2,076 Chinese controls from Singapore who share the same ancestral origin as the other Chinese controls)^5^. To boost the statistical power of the current study, we included 6,511 control subjects of Chinese Han ethnicity from several of our previous GWAS including 981 subjects from Guangdong, 523 subjects from Shandong and 5,007 subjects from Singapore. For the validation study, three independent case–control samples were recruited from China as replication 1 (2,651 IgAN cases and 2,907 controls), replication 2 (2,428 cases and 4,202 controls) and replication 3 (1,800 IgAN cases and1, 910 controls) (Supplementary Note 1).

All the cases were histopathologically diagnosed by biopsy according to the following criteria: (i) immunofluorescence showing at least 2+(scale 0 to 3+) mesangial deposition of IgA, with IgA comprising the dominant immunoglobulin deposited in the glomeruli and (ii) excluding individuals with cirrhosis, Henoch–Schönlein purpura nephritis, hepatitis B-associated glomerulonephritis, HIV infection and systemic lupus erythematosus^5^. In accordance to the Oxford Classification of IgAN, our samples were graded by the four pathological features (mesangial hypercellularity M, endocapillary hypercellularity E, segmental glomerulosclerosis S and tubular atrophy/interstitial fibrosis T, resulting in a MEST score; Supplementary Note 1)^28^,^29^.

The study was approved by the Institutional Review Board at The First Affiliated Hospital of Sun Yat-sen University and at the National University of Singapore. Written informed consent was obtained from all of the participants.

### Sample genotyping and quality control

Genomic DNA was isolated from whole blood using a Qiagen DNA extraction kit and quantified using a Picogreen assay (Invitrogen). Genotyping analysis of the discovery samples was conducted using Human660-Quad (1,523 cases), Human610-Quad (1,953 southern and 1,228 Northern Chinese controls and the 3,998 Singaporean Chinese controls, 523 Shandong controls), Human 550K (1,022 Singaporean Chinese controls), Human 1M-Duo (930 Singaporean Chinese controls) and Human OmniExpress (1,133 Singaporean Chinese controls) BeadChips (Illumina).We excluded SNPs from the X, Y and mitochondrial chromosomes and focused all further analyses on autosomal SNPs. We performed identity by descent analysis using PLINK^30^ and 104 first degree relative pairs were identified; the relative with a lower sample call rate was excluded. Principal components analysis^31^ (Eigensoft v3.0: http://genepath.med.harvard.edu/~reich/Software.htm) was done on using a set on 47,462 common SNPs (MAF>1%) that were derived from 250,201 genotyped SNPs overlapping across all arrays. These SNPs were pruned to remove SNPs in LD (*r*^2^>0.1, using PLINK --indep-pairwise 50 5 0.1) after exclusion of SNPs in the five conserved long-range LD regions in Chinese, namely the HLA region on chromosome 6, inversions on chromosomes 8 and 5 and two regions on chromosome 11 (refs. 5, 27). After principal components analysis, 16 outliers were identified based on principal components (PCs) 1–5 and excluded such that 12,095 samples (1,434 cases and 10,661 controls) were left for the final analysis.

### Genotype imputation and quality control

The software IMPUTE version 2 (https://mathgen.stats.ox.ac.uk/impute/impute_v2.html) was used for imputing genotype data of untyped SNPs in each data set following pre-phasing using SHAPEIT (https://mathgen.stats.ox.ac.uk/genetics_software/shapeit/shapeit.html)^6^,^7^,^8^,^32^. We imputed on the basis of only those genotyped SNPs that passed quality control thresholds (call rate >95%, MAF >1%, Hardy–Weinberg equilibrium (HWE) *P*>1 × 10^−6^ in controls) in each of the data sets. The imputation was performed by using the multi-ethnic 1,000-genome reference panel (dated March 2012) consisting of 1,092 individuals from Africa, Asia, Europe andthe Americas, which has been shown to outperform imputations with reference panels of matched ancestry^7^. SNP quality control criteria after imputation are as follows: MAF >1%, Impute info >0.5 if MAF ≥5%, Impute info>0.8 if MAF <5%, HWE *P* in controls >1 × 10^−8^ and average maximum posterior probabilities >0.99, leaving 3,792,915 SNPs for genotype dosage analyses in the final merged data set. For analysis of threshold-selected genotypes of 3,731,832 SNPs using GEMMA (http://www.xzlab.org/software.html)^11^,^12^, we used the following quality control filters: call rate >0.95 after setting all genotypes with probabilities <0.9 to missing, MAF >0.01, HWE *P* in controls>0.05, test of differential missingness between cases and controls *P* >1E−8, imputation info >0.8.

### HLA imputation and analysis

Imputation of two- and four-digit classical HLA alleles was performed using the SNP2HLA tool (https://www.broadinstitute.org/mpg/snp2hla/) and the Pan-Asian reference panel^13^,^14^,^15^ using the same set of directly genotyped SNPs within chr6:20–40 Mb (Hg19, build 37) that passed quality control thresholds in each of the data sets as described above. We analysed genotype dosages of all imputed HLA alleles, with most of the reported alleles having high imputation confidence (*r*^2^>0.9; Supplementary Table 4).

### Genotyping and quality controls in the validation study

Genotyping analysis of the SNPs selected for validation was performed using the MassArray system from Sequenom. Locus-specific PCR and detection primers were designed using the MassArray Assay Design 3.0 software (Sequenom). SNPs that failed Sequenom design or genotyping in validation 2 and 3 samples were genotyped using Taqman assays (Life Technologies). TaqMan reactions were carried out in 5-μl volumes containing 10–20 ng DNA according to the manufacturer's protocols. Fluorescence data were obtained in the ABI PRISM 7900HT and SDS 2.4 software (Life Technologies) was used to call genotypes. For all SNPs, we examined the clustering patterns of genotypes and selected mass peaks and confirmed that the genotype calls were of good quality. All SNPs with call rates <95% and/or HWE *P*<1 × 10^−6^ in controls and all samples with call rates of <90% were removed from further analysis from each batch (validations 1–3). After quality control, 130 SNPs from validation 1, 24 SNPs from validation 2 and 10 SNPs from validation 3 (including *CFH* SNP rs6677604) were left for further analysis.

### Association tests

Imputed dosage data were analysed using SNPTEST (https://mathgen.stats.ox.ac.uk/genetics_software/snptest/snptest.html)^10^. Genome-wide case–control analysis was performed using frequentist tests under a missing data logistic regression model, as implemented in SNPTEST. We assumed an additive model for allelic risk, with the first five principal components as covariates to control for population stratification. To evaluate the effects of mixing Northern and Southern Chinese samples in the discovery analysis, we also split the discovery cohort into two clusters based on the first two principal components^30^, one of which is predominantly Northern Chinese (414 cases and 2,306 controls), and the other predominantly Southern Chinese (1,020 cases and 8,355 controls). In each cluster, we re-ran the principal components analysis and performed the association analysis adjusted for PCs1–5 in that cluster, then meta-analysed the PC-adjusted results. The results were found to be similar and hence we kept the results from the first analysis in which we combined Northern and Southern samples. Finally, we ran Wald tests using a multilvariate linear mixed model to correct for population stratification as implemented in the package GEMMA^6^,^7^,^8^. SNPs with *P* values differing by more than three log_10_ between the two methods were excluded from further validation. Lambda 1,000 was calculated as a standardized estimate of the genomic inflation regardless of the sample size of the study^33^,^34^, using the following formula: *λ*_1,000_=1+(1−*λ*_obs_) × (1/*n*_cases_+1/*n*_controls_)/(1/1,000_cases_+1/1,000_controls_).

For the validation studies, we performed the trend test in a logistic regression model, analysing samples from Northern and Southern regions of China in validation 2 as separate sample collections to control for potential confounding by population stratification. To combine the association statistics from the GWAS and the three replication samples, we conducted a fixed-effects inverse variance meta-analysis using PLINK (http://pngu.mgh.harvard.edu/~purcell/plink/)^30^. Tests for independent association signals were carried out using conditional logistic regression analyses implemented in PLINK^30^. Haplotype analyses were conducted by phasing genotypes of interest using PHASE (http://stephenslab.uchicago.edu/software.html#phase)^35^ and analysing phased haplotypes using logistic regression analyses on PLINK^30^. Regional association plots, centred on the top SNP, were generated using LocusZoom (http://csg.sph.umich.edu/locuszoom/)^36^ to represent results from the logistic regression and fixed-effects meta-analysis at each stage of the study.

Furthermore, to evaluate the effects of age and gender on the association results, we entered age and gender as covariates into the logistic regression model and compared the results without adjustment within the same subset of samples with age and gender information available (6,323 cases and 17,349 controls; 84.6%). Each of the four stages were analysed separately and the results combined in a meta-analysis as before. Stratified analysis was performed by analysing males (3,067 cases and 9,993 controls) and females (3,324 cases and 7,371 controls) separately, among 6,391 cases and 17,364 controls with gender information available. Association results were also based on the combined meta-analysis across all four stages. Cochrane's *Q* test was used to test for heterogeneity in effect sizes between males and females, and also between Northern and Southern samples.

### Fraction of variance explained by loci

The percentage of the total variance explained was estimated by calculating Nagelkerke's pseudo R^2^ using the fmsb package (http://cran.r-project.org/web/packages/fmsb/index.html), from the result of entering SNP genotypes and affection status into the glm function in R (v 2.15.1).

## Additional information

**How to cite this article:** Li, M. *et al.* Identification of new susceptibility loci for IgA nephropathy in Han Chinese. *Nat. Commun.* 6:7270 doi: 10.1038/ncomms8270 (2015).

## Supplementary Material

Supplementary Information Supplementary Figures 1-12, Supplementary Tables 1-16, Supplementary Note 1 and Supplementary References

Supplementary Data 1 The detailed information of 115 SNPs analyzed in validation 1

Supplementary Data 2 RegulomeDB annotation of SNPs in novel and suggestive loci in LD (r2>0.8) with the IgAN-associated SNPs

Supplementary Data 3 HaploRegv2 annotation of SNPs in novel and suggestive loci in LD (r2>0.8) with the IgAN-associated SNPs

## Figures and Tables

**Table 1:** Novel SNPs reaching genome-wide significance and suggestive SNPs approaching genome wide significance.

**SNP/locus*****I***^**2**^**/*****P***_**het**_	**Risk/non-risk allele**	**Sample**	**Risk allele frequency cases (%)**	**Risk allele frequency controls (%)**	***P*** **value**	**OR (95% CI)**
rs7190997	C/T	GWAS logistic	72.0	69.2	4.00E−05	1.207 (1.103–1.320)
		GWAS gemma			1.94E−05	
chr16: 31368178		Validation 1	73.5	66.7	3.35E−15	1.386 (1.278–1.503)
*ITGAX-ITGAM*		Validation 2 Southern	71.9	69.3	3.91E−02	1.129 (1.006–1.267)
		Validation 2 Northern	77.3	76.2	3.01E−01	1.064 (0.946–1.196)
		Validation 3 Southern	72.9	68.4	1.16E−03	1.239 (1.089–1.411)
		Validation 3 Northern	77.5	74.7	7.30E−02	1.166 (0.986–1.379)
		All validation			1.10E−15	1.229 (1.168–1.292)
		Meta-analysis	*I*^2^=70.3%, *P*_het_=0.0049		2.26E−19	1.223 (1.171–1.278)
						
rs2074038	T/G	GWAS logistic	32.3	28.8	3.36E−05	1.207 (1.104–1.319)
		GWAS gemma			4.45E−05	
chr11:44087989		Validation 1	31.3	28.6	1.87E−03	1.137 (1.049–1.233)
*ACCS*		Validation 2 Southern	32.5	28.9	4.08E−03	1.177 (1.053–1.315)
		Validation 2 Northern	34.8	33.6	3.24E−01	1.054 (0.950–1.170)
		Validation 3 Southern	30.4	29.1	3.42E−01	1.066 (0.935–1.216)
		Validation 3 Northern	35.6	32.7	1.03E−01	1.134 (0.975–1.318)
		All validation			8.76E−06	1.116 (1.063–1.171)
		Meta-analysis	*I*^2^=1.26%, *P*_het_=0.408		3.93E−09	1.136 (1.089–1.185)
						
rs2033562	C/G	GWAS logistic	51.4	47.7	7.98E−05	1.176 (1.085–1.274)
		GWAS gemma			4.14E−04	
chr8:103547739		Validation 1	50.5	47.7	3.45E−03	1.116 (1.037–1.201)
*KLF10/ODF1*		Validation 2 Southern	50.6	48.8	1.77E−01	1.074 (0.968–1.192)
		Validation 2 Northern	50.8	47.4	9.15E−03	1.139 (1.033–1.256)
		Validation 3 Southern	49.8	49.0	5.90E−01	1.033 (0.918–1.163)
		Validation 3 Northern	50.9	45.0	1.35E−03	1.265 (1.096–1.460)
		All validation			2.21E−06	1.114 (1.065–1.165)
		Meta-analysis	*I*^2^=23.7%, *P*_het_=0.256		1.41E−09	1.128 (1.085–1.173)
						
rs7634389	C/T	GWAS logistic	47.4	43.5	6.80E−04	1.151 (1.061–1.248)
		GWAS gemma			4.33E−05	
chr3:186738421		Validation 1	46.2	43.7	8.86E−03	1.105 (1.026–1.191)
*ST6GAL1*		Validation 2 Southern	46.1	44.3	1.80E−01	1.075 (0.968–1.194)
		Validation 2 Northern	46.6	42.6	1.48E−03	1.176 (1.064–1.300)
		Validation 3 Southern	47.0	42.8	5.94E−03	1.180 (1.049–1.328)
		Validation 3 Northern	46.7	43.8	1.09E−01	1.124 (0.974–1.297)
		All validation			2.48E−07	1.126 (1.076–1.178)
		Meta-analysis	*I*^2^=0%, *P*_het_=0.772		7.27E−10	1.132 (1.088–1.178)

CI, confidence interval; GWAS, genome-wide association study; OR, odds ratio; SNP, single-nucleotide polymorphism.

For the GWAS samples, only the result from logistic regression analyses (GWAS logistic) was used in the fixed-effects meta-analysis with the validation samples. *P* values shown are from logistic regression analyses unless otherwise stated.

**Table 2:** Three independent signals in the *DEFA* region. *r* ^2^=0 between rs9314614 and rs12716641.

**SNP**	**Risk allele**	**Risk allele**			Multivariate analysis*	
**Risk/non-risk allele**	**frequency cases (%)**	**frequency controls (%)**	***P*** **value**	**OR (95% CI)**	***P*** **value**	**OR (95% CI)**
**rs2738058**	**chr8:6821617**					
T/C						
GWAS logistic	73.0	66.6	5.75E−10	1.310 (1.203–1.427)	2.06E−08	1.298 (1.185–1.422)
GWAS gemma			1.51E−10			
Validation 1	72.4	69.0	6.10E−05	1.181 (1.089–1.281)	4.88E−03	1.131 (1.038–1.232)
Validation 2 Southern	71.9	66.0	1.92E−06	1.327 (1.181–1.491)	6.46E−05	1.284 (1.136–1.451)
Validation 2 Northern	72.3	67.6	7.08E−05	1.245 (1.117–1.386)	6.28E−04	1.219 (1.088–1.366)
Validation 3 Southern	72.6	67.3	8.74E−05	1.303 (1.142–1.487)	8.58E−04	1.263 (1.101–1.450)
Validation 3 Northern	73.4	67.0	1.64E−04	1.354 (1.156–1.585)	1.26E−03	1.310 (1.112–1.543)
All validation			4.14E−19	1.253 (1.193–1.317)	1.67E−17	1.226 (1.170–1.285)
Meta-analysis	*I*^2^=31%, *P*_het_=0.400		2.31E−27	1.267 (1.214–1.323)	1.15E−19	1.232 (1.178–1.289)
						
**rs9314614**	**chr8:6697731**					
C/G						
GWAS logistic	39.7	36.0	3.54E−05	1.191 (1.097–1.295)	2.85E−04	1.161 (1.071–1.258)
GWAS gemma			6.20E−05			
Validation 1	37.7	35.0	3.77E−03	1.121 (1.038–1.211)	2.03E−03	1.130 (1.046–1.222)
Validation 2 Southern	38.6	35.6	2.10E−02	1.135 (1.019–1.264)	3.03E−02	1.127 (1.011–1.256)
Validation 2 Northern	40.2	39.1	3.42E−01	1.051 (0.948–1.165)	3.72E−01	1.048 (0.945–1.163)
Validation 3 Southern	38.4	36.0	1.03E−01	1.108 (0.980–1.253)	9.97E−02	1.109 (0.981–1.255)
Validation 3 Northern	41.9	36.8	4.85E−03	1.234 (1.066–1.428)	6.73E−03	1.225 (1.058–1.419)
All validation			2.66E−06	1.118 (1.067–1.171)	9.55E−08	1.121 (1.075–1.170)
Meta-analysis	*I*^2^=0%, *P*_het_=0.442		9.48E−10	1.135 (1.090–1.182)	4.25E−09	1.129 (1.084–1.176)
						
**rs12716641**	**chr8:6898998**					
T/C						
GWAS logistic	78.0	74.3	7.80E−07	1.260 (1.150–1.381)	4.40E−03	1.153 (1.045–1.272)
GWAS gemma			1.79E−06			
Validation 1	78.2	73.6	1.22E−08	1.287 (1.180–1.404)	2.95E−06	1.244 (1.135–1.363)
Validation 2 Southern	78.8	75.2	1.81E−03	1.219 (1.076–1.381)	8.47E−02	1.122 (0.984–1.278)
Validation 2 Northern	80.4	78.4	5.44E−02	1.129 (0.998–1.278)	3.91E−01	1.058 (0.929–1.205)
Validation 3 Southern	78.7	75.5	1.00E−02	1.204 (1.045–1.386)	1.01E−01	1.131 (0.976–1.311)
Validation 3 Northern	80.5	77.4	3.85E−02	1.207 (1.010–1.442)	2.65E−01	1.112 (0.923–1.339)
All validation			2.34E−13	1.224 (1.159–1.291)	1.66E−08	1.157 (1.100–1.218)
Meta-analysis	*I*^2^=0%, *P*_het_=0.432		1.13E−18	1.233 (1.177–1.292)	9.53E−09	1.154 (1.099–1.212)

CI, confidence interval; GWAS, genome-wide association study; OR, odds ratio; SNP, single-nucleotide polymorphism.

Only rs2738058 is in LD (*r*^2^=0.71) with the reported SNP rs2738048.For the GWAS samples, only the result from logistic regression analyses (GWAS logistic) was used in the fixed-effects meta-analysis with the validation samples.

^*^Pairwise LD (*r*^2^) between rs2738058 and rs9314614=0.001, between rs12716641 and rs2738058=0.074. There is no LD between rs9314614 and rs12716641 (*r*^2^=0).

**Table 3:** Functional annotations of novel loci and associated SNPs (details on eQTLs and ENCODE annotations in Supplementary Tables 14 and 15, Supplementary Data 2 and 3)^16^,^21^,^22^,^23^,^24^,^25^,^37^,^38^,^39^,^40^,^41^,^42^,^43^.

**SNP/locus**	**eQTL/ENCODE**	**Other diseases**	**Known functions of genes**
rs207403811p11.2SNP location: Lies within either the first intron or 5′ UTR of the various *ACCS/PHACS* gene isoformsWithin the same LD block as the adjacent gene *EXT2*.	Risk allele is strongly associated with increased *ACCS* gene expression levels in peripheral blood cells (*P*<9.81 × 10^−198^), monocytes (*P*=1.07 × 10^−36^), and B-cells (*P*=1.46 × 10^−29^).It is also associated with increased *EXT2* expression levels in peripheral blood cells (*P*=2.63 × 10^−53^).This SNP and others in LD are also located at sites predicted with high likelihood to affect chromatin structure and protein binding.	Not previously associated with any other trait.	*ACCS* encodes a1-aminocyclopropane-1-carboxylate synthase homologue that belongs to the class-I pyridoxal-phosphate-dependent aminotransferase family.Shown to interact with the protein encoded by *FBF1* (Fas (TNFRSF6) binding factor 1), a keratin-binding protein that is required for epithelial cell polarization, apical junction complex assembly andciliogenesis^37^,^38^*EXT2* is a glycosyltransferase, which plays a role in heparan sulfate biosynthesis. Heparan sulfate in turn influences angiogenesis and cell proliferation; mutations in this gene cause multiple exotoses^39^,^40^.
rs76343893q27.3SNP location: Lies within an intron of the *ST6GAL1*.	Risk allele is in strong LD (*r*^2^>0.9 in our samples, HapMap Europeans and Asians) with variants associated with decreased expression levels of *ST6GAL1* in peripheral blood cells (rs3821819; *P*=5.96 × 10^−20^) and B-cells (rs17776120; *P*=1.61 × 10^−7^).This SNP and others in LD may affect chromatin structure and protein binding.	IgG glycosylation, Type 2 diabetes, oesophageal cancer.Drug-induced liver injury.In moderate LD with rs11710456 in IgG glycosylation in Europeans (*r*^2^=0.528) but not Asians (*r*^2^=0.12).	*ST6GAL1* encodes ST6 beta-galactosamide alpha-2,6-sialyltranferase, a member of glycosyltransferase family involved in the generation of the cell-surface carbohydrate determinants and differentiation antigens.Regulates macrophage apoptosis via alpha2-6 sialylation of the TNFR1 death receptor.May play a regulatory role in innate immune response.Up-regulated in human cancers^41^,^42^.
rs20335628q22.3SNP location: Located in an intergenic region closest to the genes *ODF1, KLF10* and *UBR5*.	No evidence for effects on gene expression levels in peripheral blood cells, monocytes, B-cells.In LD with SNPs with potential regulatory effects on *UBR5* expression, chromatin structure and protein binding.	Chronic lymphocytic leukaemia (CLL)Not in LD with CLL-associated SNP.	*KLF10* encodes a transcriptional repressor that acts as an effector of transforming growth factor beta signalling and activity of this protein may inhibit the growth of cancers.*UBR5* encodes a E3 ubiquitin ligase which interacts with the deubiquitinase DUBA which in turns plays a role in IL-17 production in T-cells and inflammatory response in the small intestine^43^.

SNP, single-nucleotide polymorphism.

Other recently and previously reported loci are described in Supplementary Table 13.
