# Supplementary material for: Identification of new susceptibility loci for IgA nephropathy in Han Chinese
Source: Nat Commun. 2015 Jun 1;6:7270. doi: 10.1038/ncomms8270 (PMC4458882; doi:10.1038/ncomms8270)
Supplement: Supplementary Information — Supplementary Figures 1-12, Supplementary Tables 1-16, Supplementary Note 1 and Supplementary References [file ncomms8270-s1.pdf]

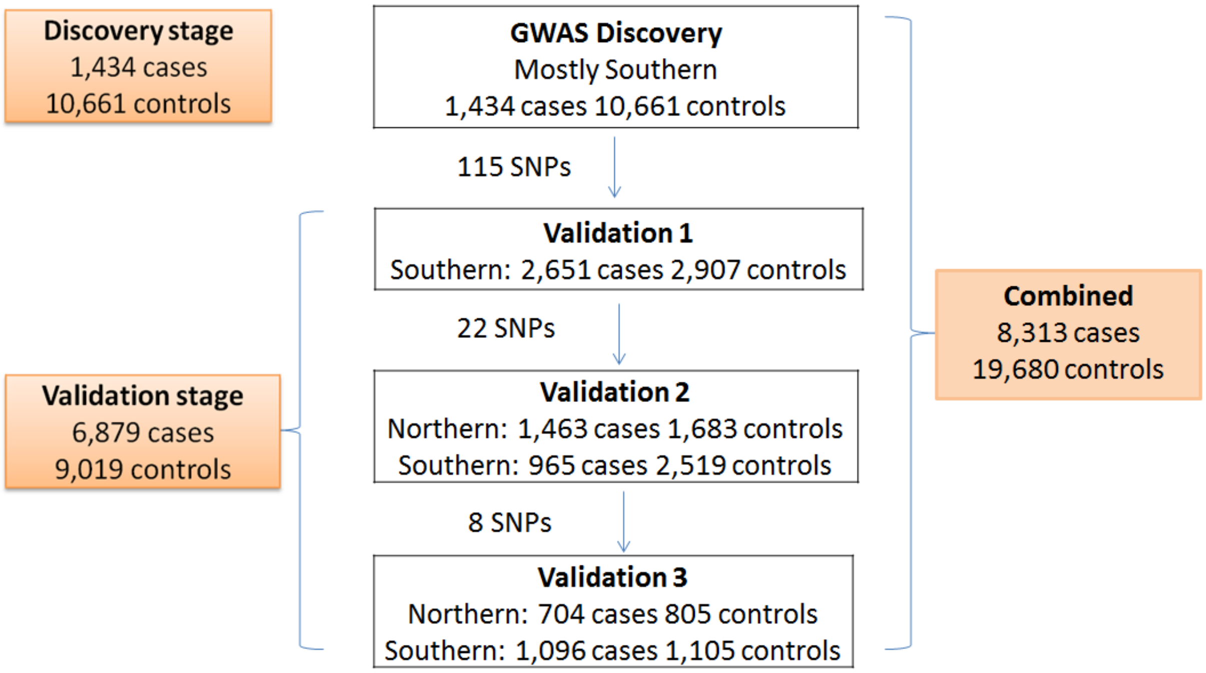

Supplementary figure 1. Flowchart showing study design and number of SNPs and samples analysed across four stages.

a)

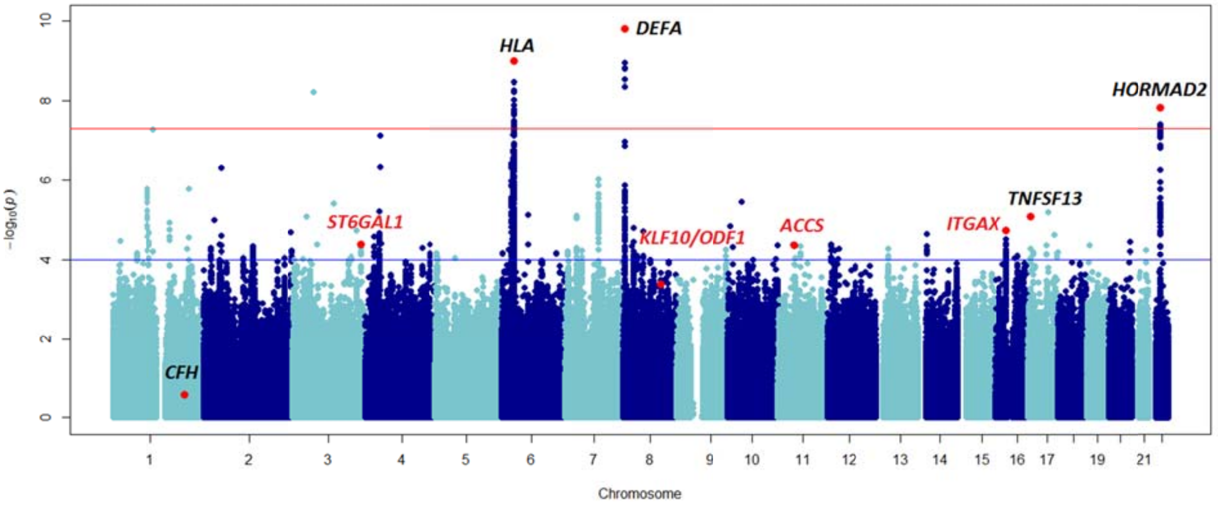

b)

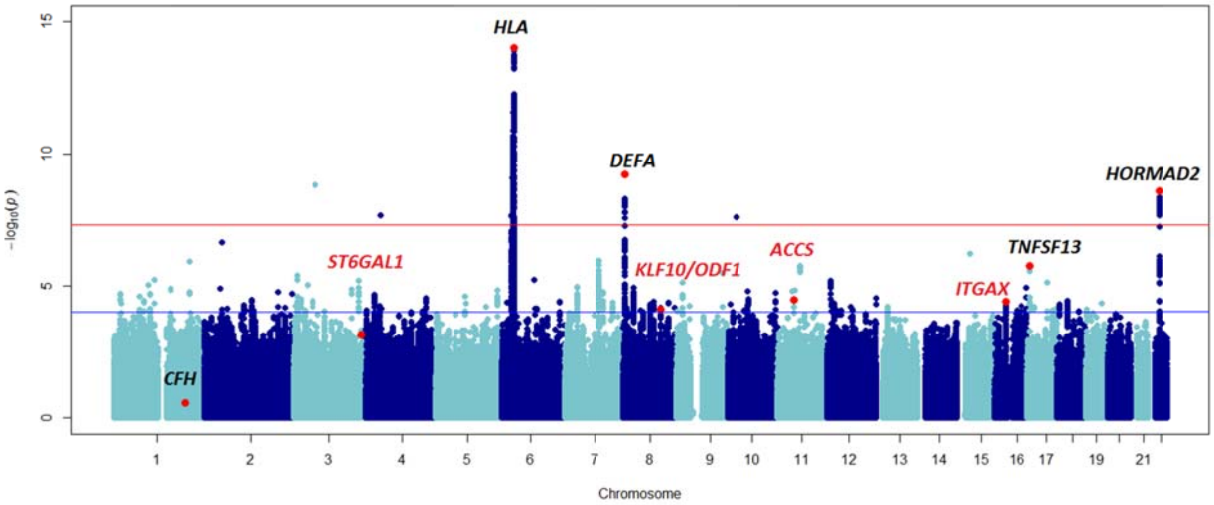

Supplementary figure 2. Manhattan plots from the genome-wide discovery stage generated using P-values of SNPs passing quality control filters from the following statistical tests: a) GEMMA[1] wald test on threshold-filtered genotypes (3.7 million SNPs) and b) Principal components (PC)-adjusted logistic regression test on genotype dosages (3.8 million SNPs). Reported SNPs within previously identified and novel loci are indicated in red.

a) λ_{GC} = 1.039, λ_{GC} without MHC SNPs= 1.035

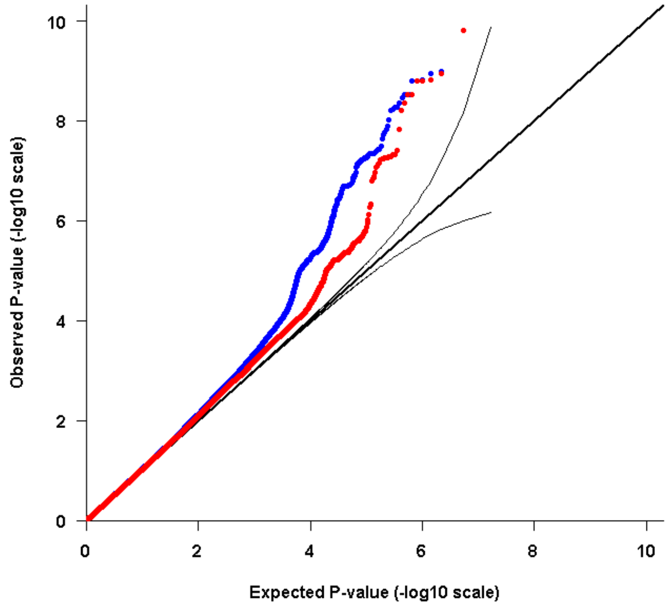

b) λ_{GC} = 1.087, λ_{GC} without MHC SNPs= 1.077

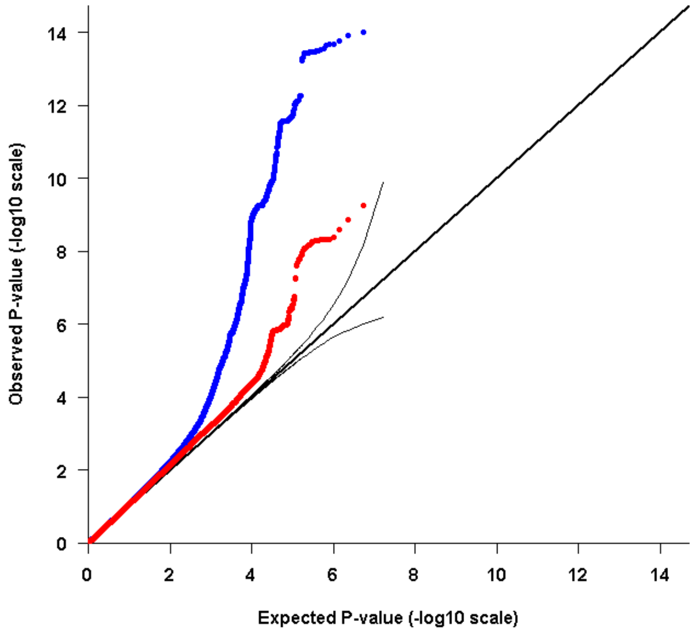

Supplementary figure 3. Quantile-quantile plots before (blue) and after (red) removing MHC SNPs from the genome-wide discovery stage showing observed vs expected P-values of SNPs passing quality control filters from the following statistical tests a) GEMMA[1] wald test on threshold-filtered genotypes and b) PC-adjusted logistic regression test on genotype dosages. The 95% confidence intervals of the P-value distributions and the genomic inflation lambda GC values are indicated.

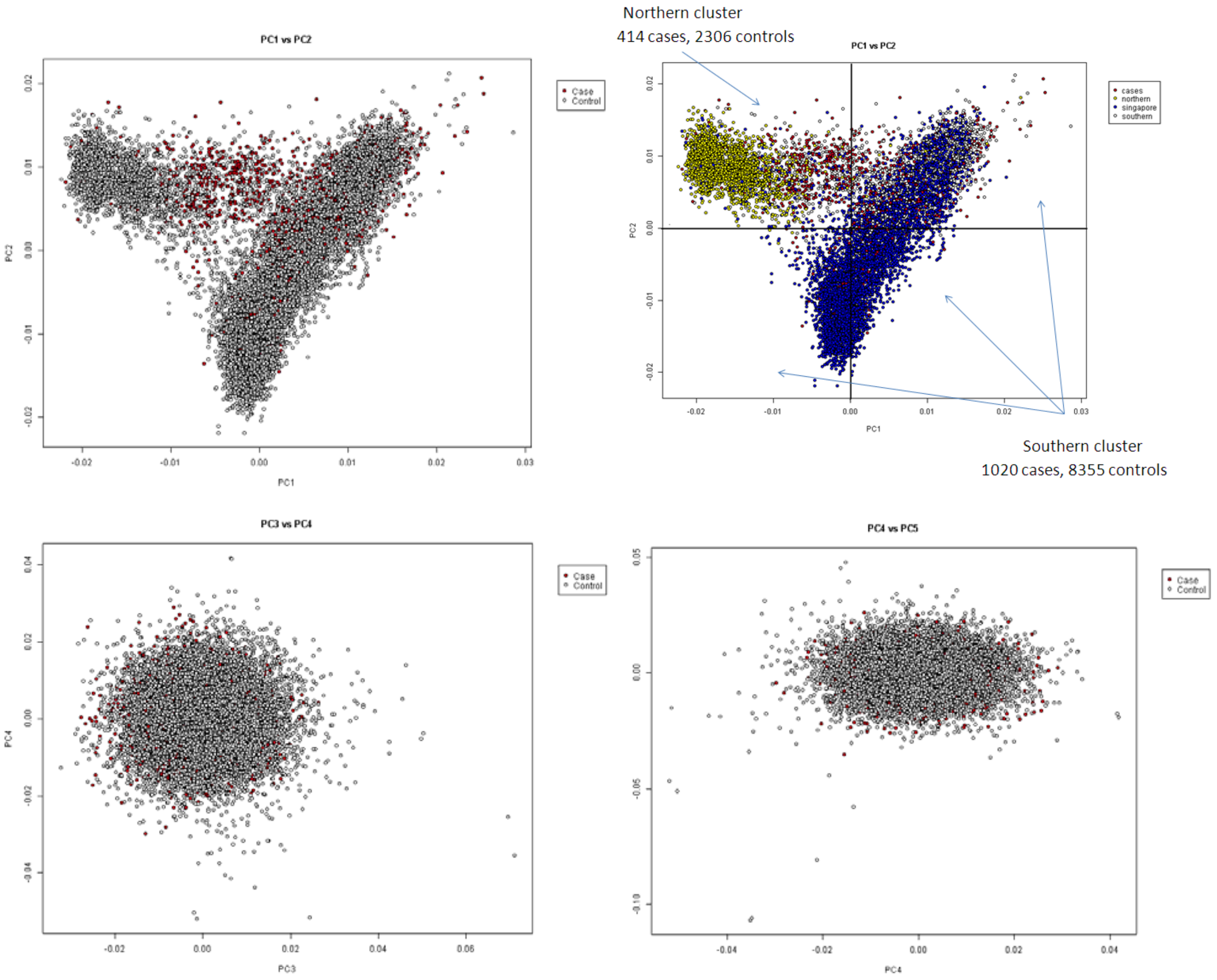

Supplementary figure 4. Principal components analysis on all samples

Plots of the first five principal components (PCs; PC1 vs PC2, PC3 vs PC4 and PC4 vs PC5), which were derived from the PCA analysis of 12,095 samples of the discovery analysis, of which 1,434 cases and 4,270 controls have been analysed in the previous GWAS[2]

Northern cluster PC1 vs 2

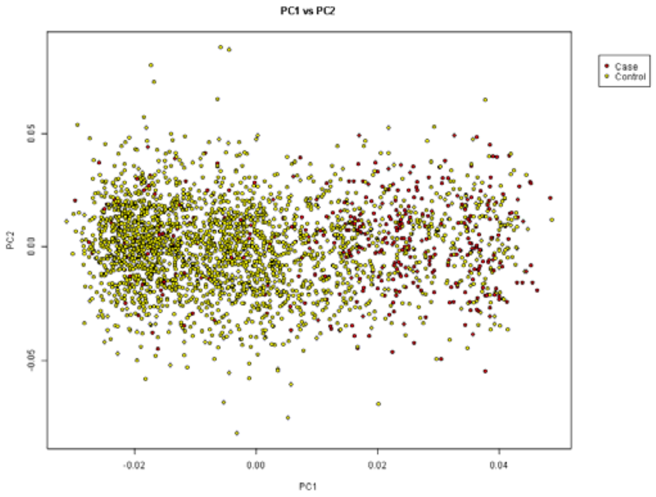

Southern Cluster PC1vs 2

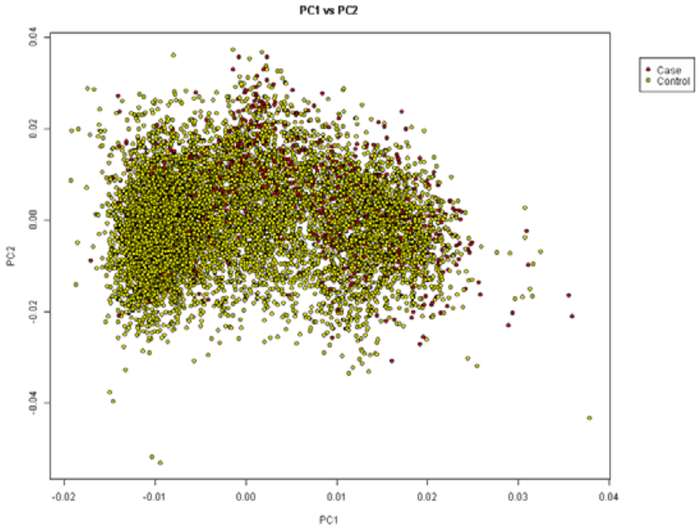

Supplementary figure 5. Principal components analysis on Northern and Southern clusters
Plots of the first two principal components (PCs; PC1 vs PC2) calculated separately for the Northern (2,581 cases, 4,794 controls) and Southern (5,732 cases, 14,886 controls) samples.

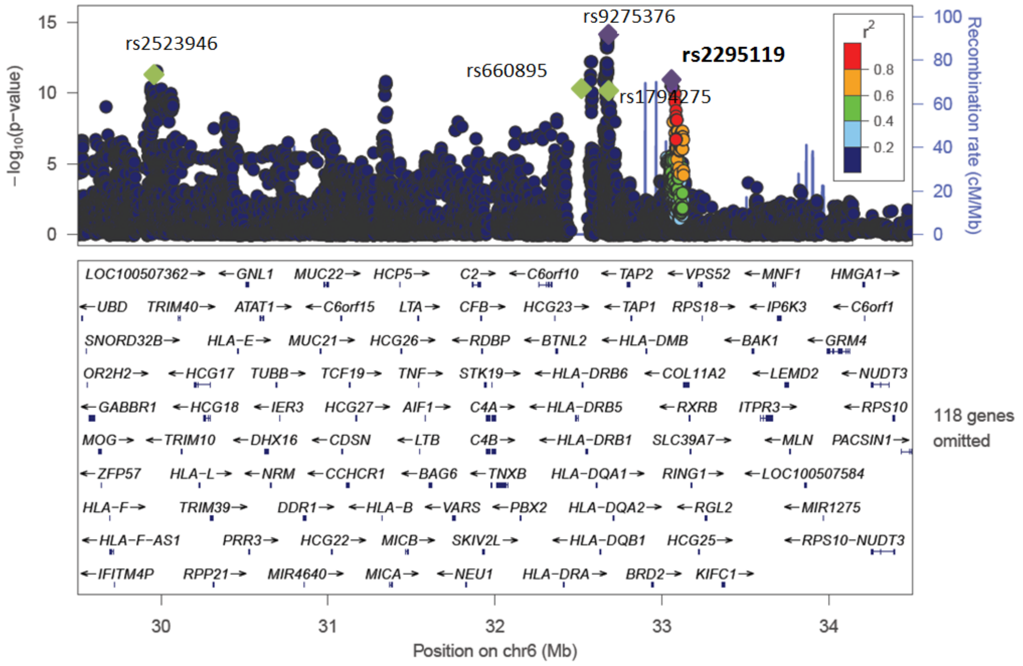

Supplementary figure 6. Regional association plot of the HLA region showing the novel independent signal (purple) and the previously reported SNPs[2] (green). The SNP rs9275376 is in LD with rs660895 (r^2=0.477, D'=0.755) and rs1794275 (r^2=0.293, D'=0.572) and was not imputed in our previous analysis because it was absent from the older reference panels (HapMap II+III). P-values shown are based on logistic regression analyses adjusted for the first five principal components.

HORMAD2

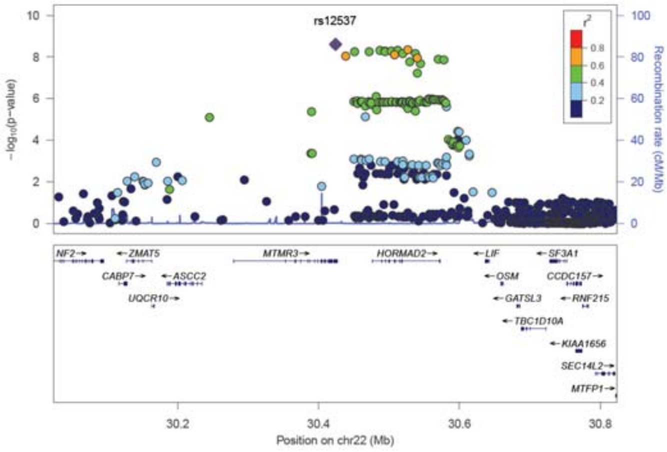

CFH

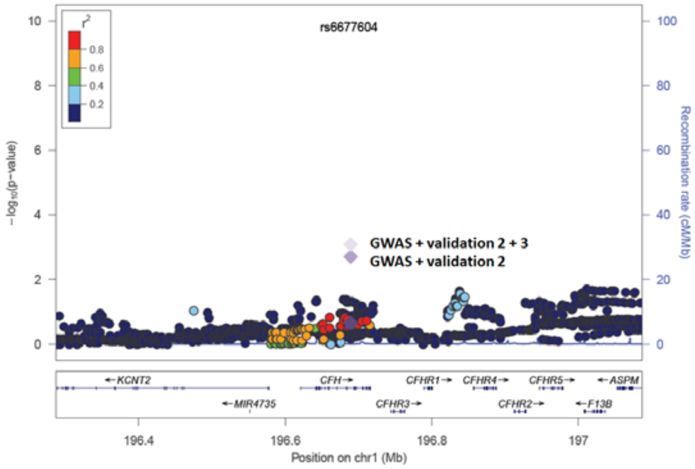

TNFSF13

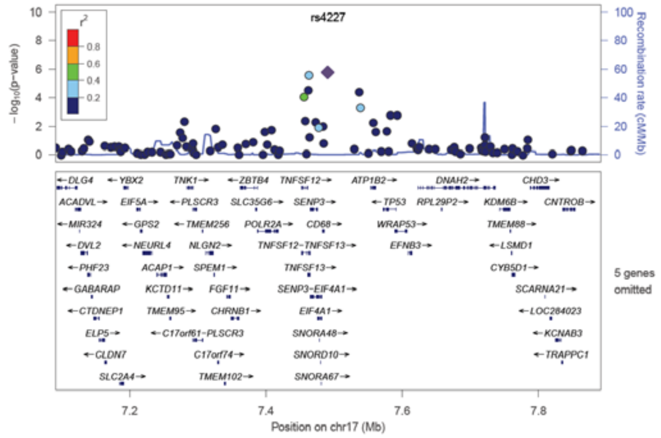

Supplementary figure 7. Regional association plots of previously reported loci[2,4]

CARD9

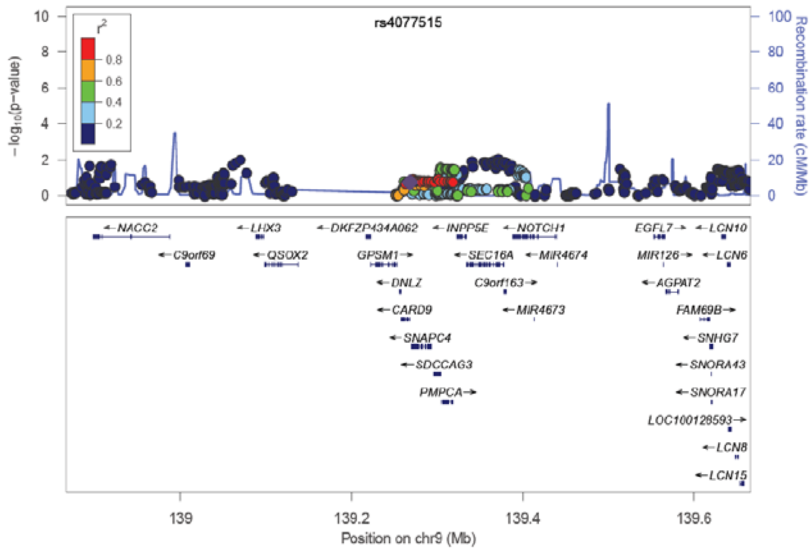

VAV3

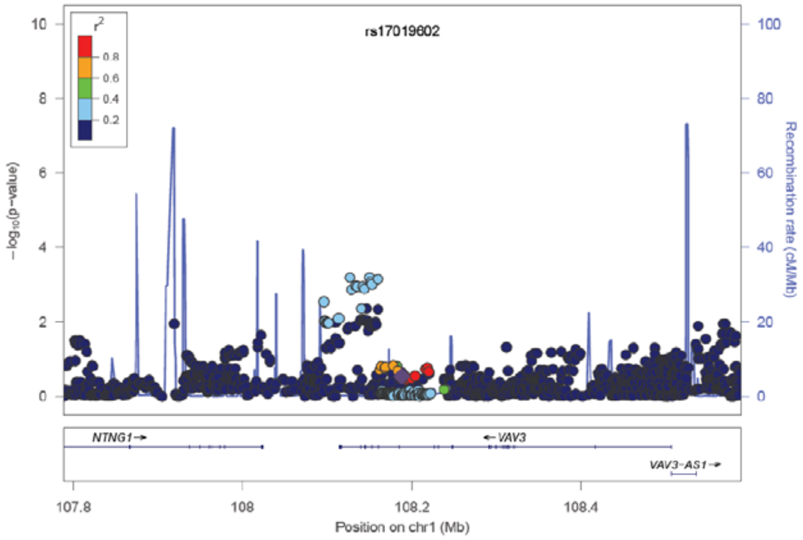

Supplementary figure 8. Regional association plots of recently reported loci[5]

a)

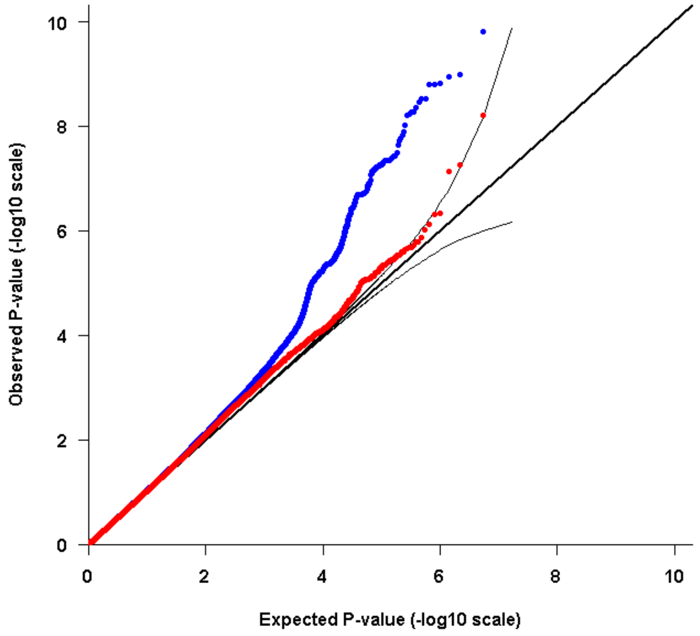

b)

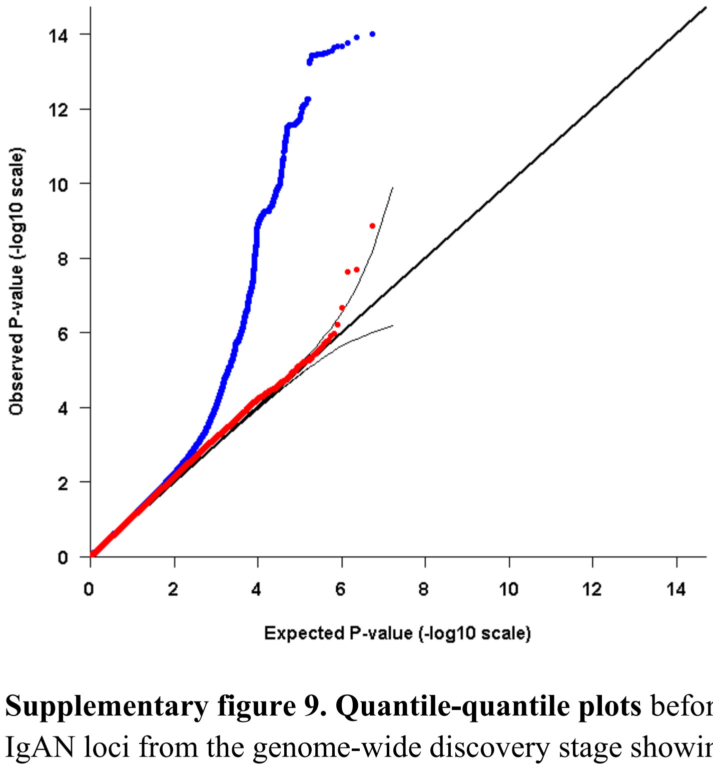

Supplementary figure 9. Quantile-quantile plots before (blue) and after (red) removing five known IgAN loci from the genome-wide discovery stage showing observed vs expected P-values of SNPs passing quality control filters from the following statistical tests: a) GEMMA[1] wald test on threshold-filtered genotypes and b) PC-adjusted logistic regression test on genotype dosages. The 95% confidence intervals of the P-value distributions are indicated.

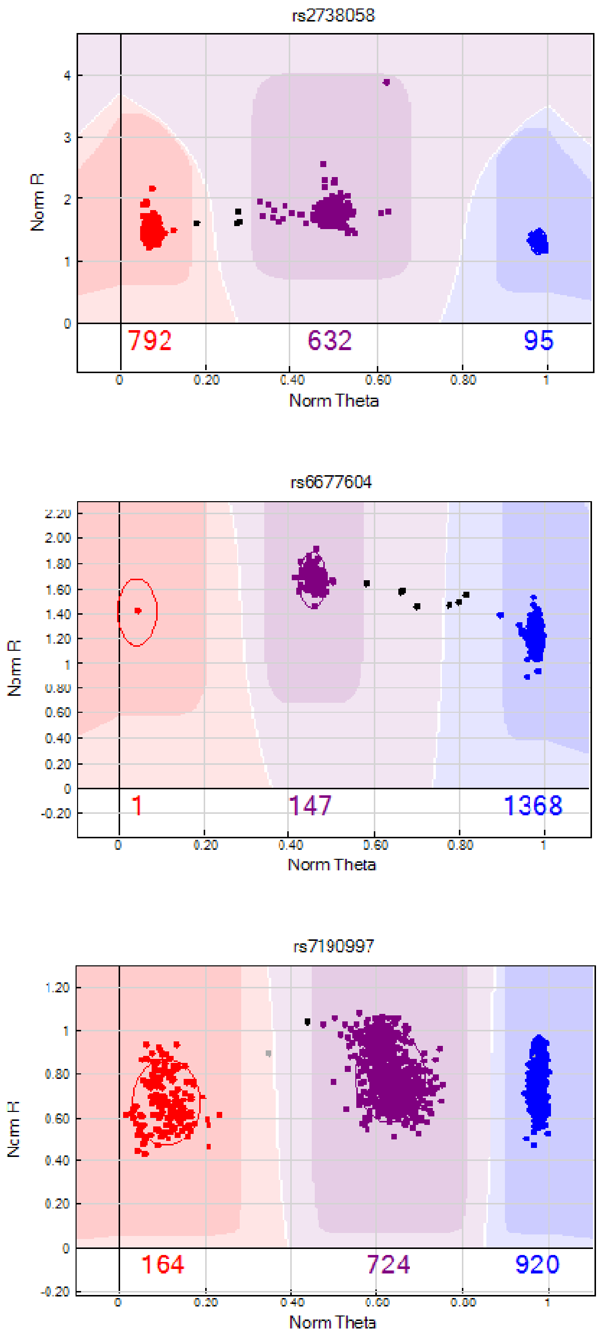

Supplementary figure 10. Examples of genotyping cluster plots of the top reported SNPs on Illumina

rs12716641

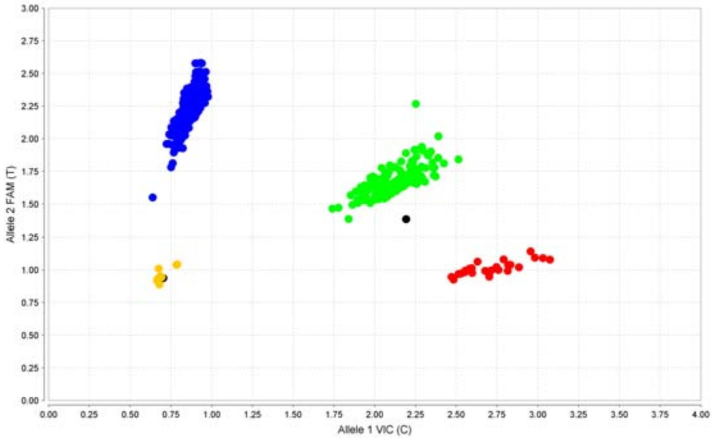

Supplementary figure 11. Example of genotyping cluster plot of rs12716641on Taqman.

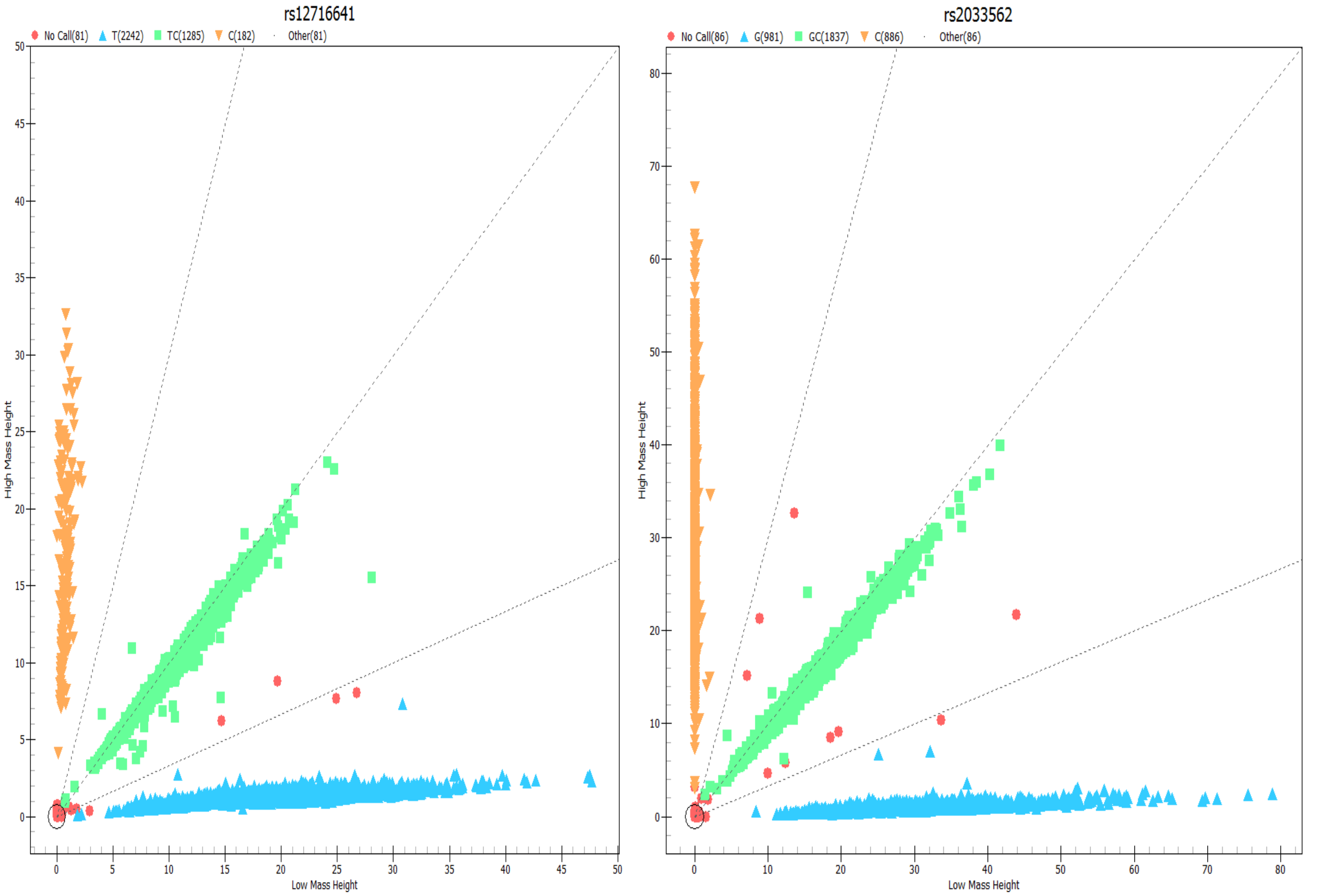

Supplementary figure 12 a). Examples of genotyping cluster plots of the top reported SNPs on Sequenom.

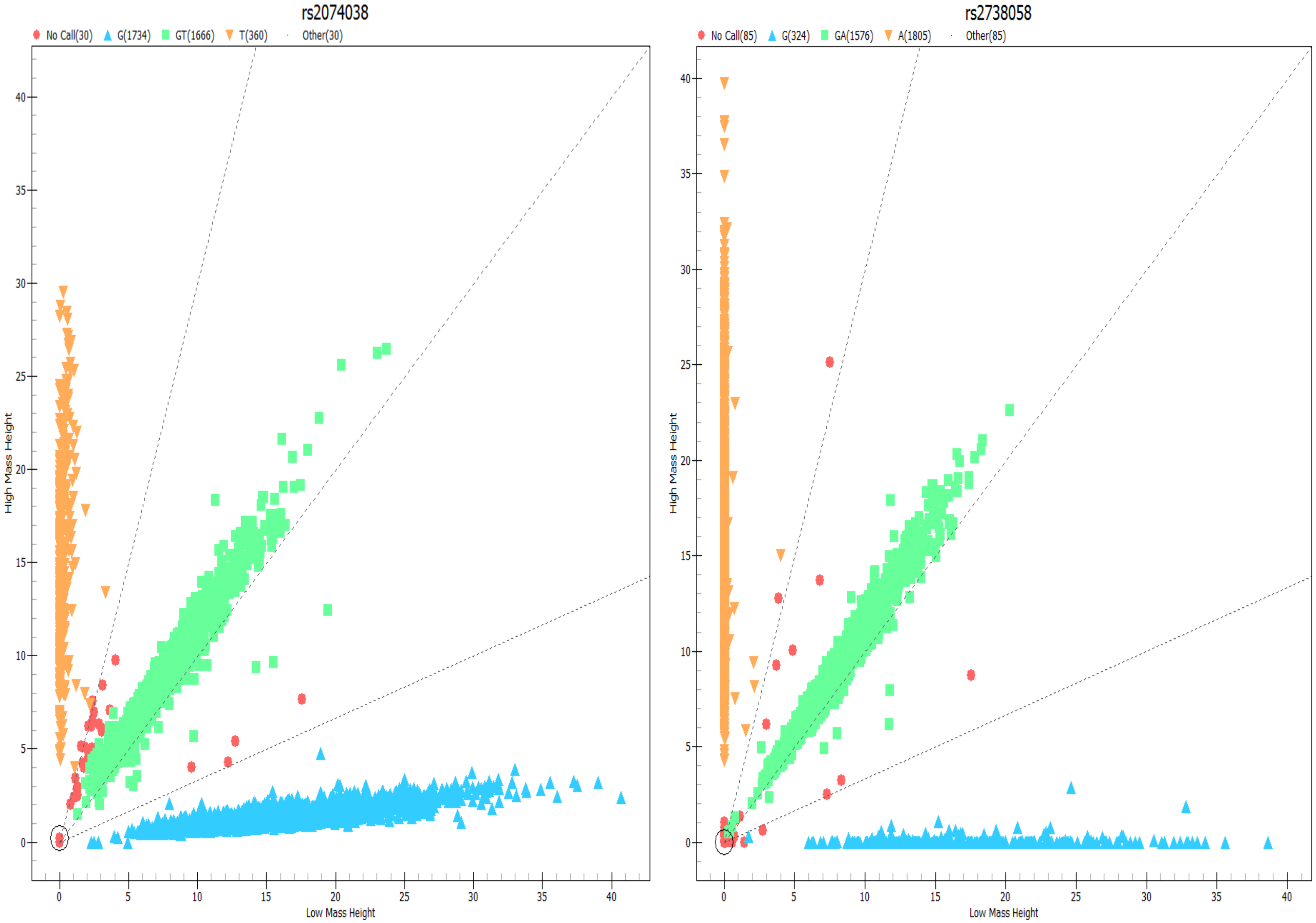

Supplementary figure 12 b). Examples of genotyping cluster plots of the top reported SNPs on Sequenom.

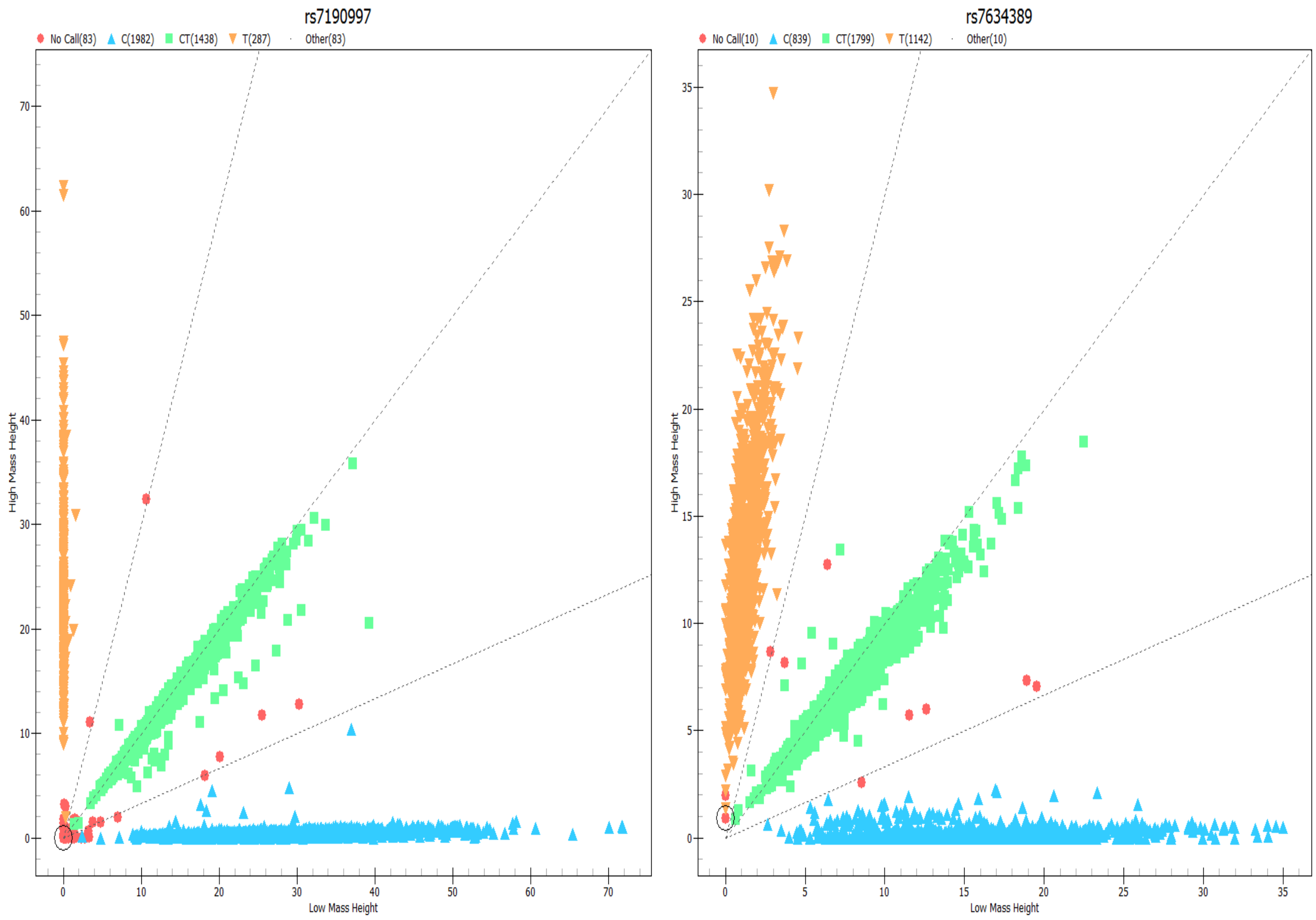

Supplementary figure 12 c). Examples of genotyping cluster plots of the top reported SNPs on Sequenom.

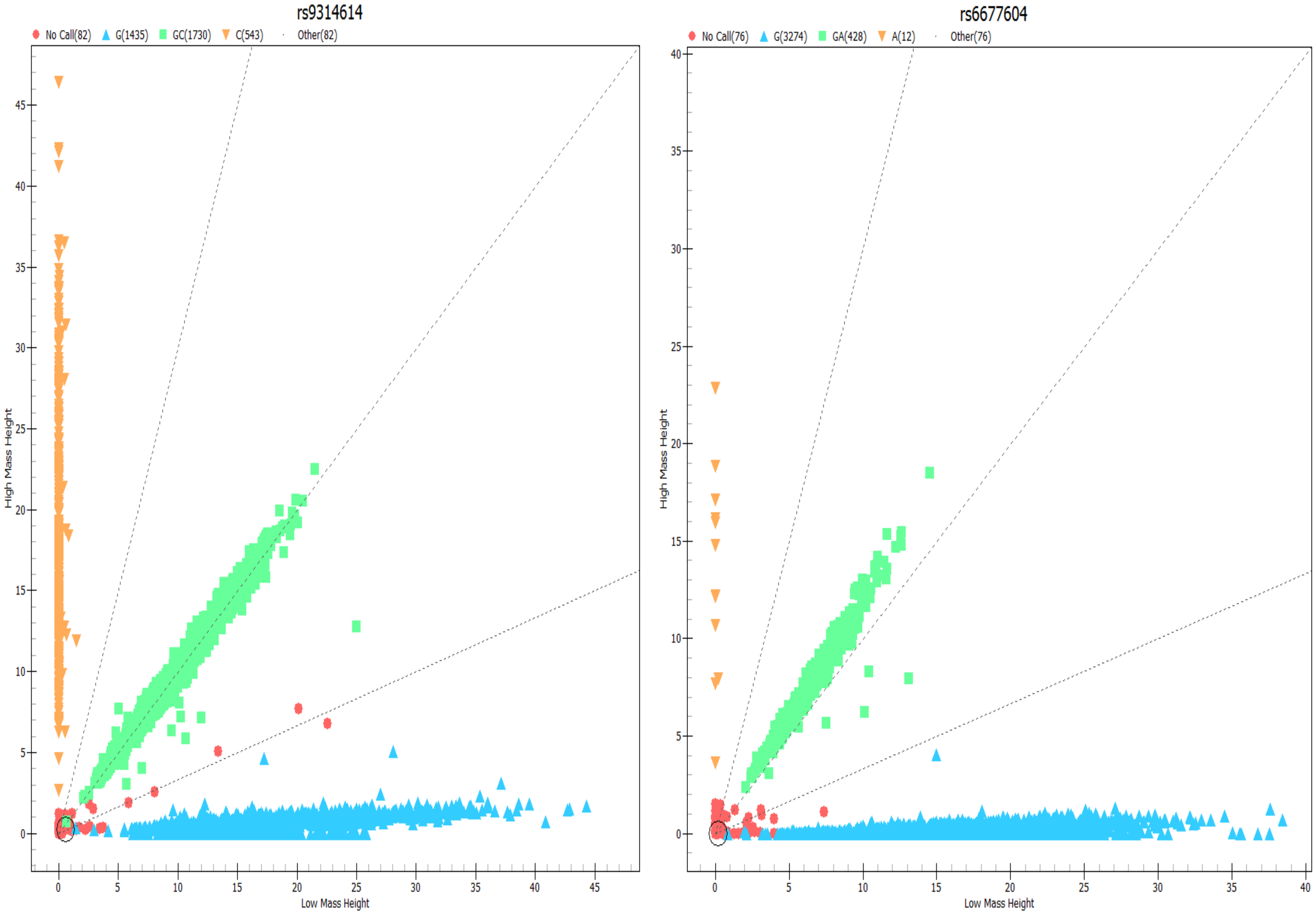

Supplementary figure 12 d). Examples of genotyping cluster plots of the top reported SNPs on Sequenom.

Cohort	Population	IgAN Cases			Controls		
		Sample size	Mean age (years)	Male/Female (%)	Sample size	Mean age (years)	Male/Female (%)
GWAS		1434#	35	44.0/56.0	4270#	43.8	47.5/52.5
					+		
					6391	44.0	57.8/42.2
Validation 1	Southern	2651	35.3±10.9	45.3/54.7	2907	38.8±13.1	65.4/34.6
Validation 2	Southern	965	35.6±12.0	52.1/47.9	2519	35.2±11.7	61.1/38.9
	Northern	1463	33.4±11.7	52.9/47.1	1683	28.9±7.6	52.4/47.6
Validation 3	Southern	1096	35.4±11.6	47.8/52.2	1105	35.9±11.2	48.8/51.2
	Northern	704	35.6±12.5	48.9/51.1	805	39.0±12.1	54.9/45.1

Supplementary table 1. Sample information. More description is available in Supplementary note.
Samples analysed in the previous GWAS[2]

Current GWAS discovery, 1,434 cases, 10,661 controls

α =0.0001, unselected controls

Minor allele frequency	OR								
	**1.1**	**1.15**	**1.2**	**1.25**	**1.3**	**1.35**	**1.4**	**1.45**	**1.5**
**0.05**	0.3%	1.2%	4.1%	11.1%	24.2%	42.5%	62.5%	79.5%	90.7%
**0.1**	0.8%	4.7%	16.9%	40.0%	67.1%	87.1%	96.5%	99.3%	99.9%
**0.2**	2.7%	16.4%	48.1%	80.5%	96.0%	99.6%	100.0%	100.0%	100%
**0.3**	4.9%	27.6%	66.9%	92.5%	99.2%	100.0%	100%	100%	100%
**0.4**	6.4%	34.1%	74.6%	95.5%	99.6%	100.0%	100%	100%	100%
**0.5**	6.7%	35.1%	75.2%	95.5%	99.6%	100.0%	100%	100%	100%

Previous GWAS discovery, 1,434 cases, 4,270 controls[2]

α =0.0001, unselected controls

Minor allele frequency	OR								
	**1.1**	**1.15**	**1.2**	**1.25**	**1.3**	**1.35**	**1.4**	**1.45**	**1.5**
**0.05**	0.2%	0.8%	2.7%	7.2%	15.9%	29.3%	46.2%	63.6%	78.4%
**0.1**	0.6%	3.1%	11.2%	28.1%	51.8%	74.5%	89.7%	96.8%	99.3%
**0.2**	1.8%	11.1%	35.3%	67.2%	89.4%	97.9%	99.7%	100.0%	100%
**0.3**	3.3%	19.4%	53.2%	84.0%	97.0%	99.7%	100%	100%	100%
**0.4**	4.3%	24.5%	61.6%	89.3%	98.4%	99.9%	100%	100%	100%
**0.5**	4.6%	25.6%	62.7%	89.6%	98.5%	99.9%	100%	100%	100%

Current GWAS combined 8,313 cases, 19,680 controls

(α =0.0001 in stage 1) x (α = 0.0001 in stage 1+2) x (α = $5x10^{-8}$) in stages 1-4 combined, unselected controls

Minor allele frequency	OR								
	**1.1**	**1.15**	**1.2**	**1.25**	**1.3**	**1.35**	**1.4**	**1.45**	**1.5**
**0.05**	0.0%	0.0%	0.2%	3.9%	18.5%	40.7%	62.2%	79.4%	90.7%
**0.1**	0.0%	0.4%	9.9%	38.0%	67.0%	87.1%	96.5%	99.3%	99.9%
**0.2**	0.1%	9.6%	47.1%	80.4%	96.0%	99.6%	100%	100%	100%
**0.3**	0.4%	23.5%	66.8%	92.5%	99.2%	100%	100%	100%	100%
**0.4**	1.0%	31.4%	74.5%	95.5%	99.6%	100%	100%	100%	100%
**0.5**	1.2%	32.7%	75.2%	95.5%	99.6%	100%	100%	100%	100%

Supplementary table 2. Calculations of statistical power (http://pngu.mgh.harvard.edu/~purcell/gpc/) in the current and previous discovery GWAS, and in the total combined samples of the current study.[3]

chromosome	SNP	position	Frequency cases	Frequency controls	Risk allele	OR (95% CI)	P_gemma	P_logistic
**Gharavi et al 2011[4]**								
1	rs3766404	196651832	92.7%	92.4%	T	1.046 (0.901-1.215)	6.86E-01	7.38E-01
1	rs6677604	196686918	95.0%	94.4%	G	1.125 (0.941-1.345)	2.64E-01	2.60E-01
6	rs2856717	32670308	83.7%	80.9%	G	1.213 (1.092-1.347)	1.59E-01	7.21E-03
6	rs9275596	32681631	89.7%	85.2%	T	1.502 (1.325-1.703)	1.16E-04	4.16E-07
6	rs9357155	32809848	86.1%	83.4%	G	1.236 (1.105-1.383)	2.34E-02	3.82E-03
6	rs2071543	32811629	85.2%	82.2%	G	1.247 (1.118-1.391)	2.17E-02	2.23E-03
6	rs3129269	33097614	75.6%	74.0%	C	1.085 (0.991-1.188)	7.88E-02	1.35E-02
6	rs1883414	33086448	79.8%	78.7%	G	1.073 (0.974-1.182)	2.59E-01	5.42E-02
22	rs2412971	30494371	75.6%	70.4%	G	1.298 (1.186-1.420)	4.77E-08	5.24E-09
22	rs2412973	30529631	74.7%	69.7%	C	1.281 (1.172-1.400)	1.34E-07	1.65E-08
**Yu et al 2012[2]**								
8	rs2738048	6822785	73.5%	67.5%	A	1.326 (1.215-1.447)	9.75E-10	5.13E-09
17	rs3803800	7462969	42.0%	37.2%	A	1.226 (1.132-1.327)	5.42E-05	2.67E-06
17	rs4227	7491177	27.3%	22.5%	G	1.298 (1.188-1.418)	8.22E-06	1.77E-06
22	rs12537	30423460	86.3%	81.7%	C	1.407 (1.258-1.573)	1.45E-08	2.54E-09
6	rs2523946	29941943	57.9%	51.1%	C	1.317 (1.217-1.426)	2.86E-07	4.54E-11
6	rs660895	32577380	31.1%	25.9%	G	1.289 (1.184-1.402)	8.02E-06	1.15E-10
6	rs1794275	32671248	18.2%	14.1%	A	1.365 (1.232-1.513)	5.23E-07	1.27E-09
**Kiryluk et al 2014[5]**								
1	rs17019602	108188858	19.5%	18.9%	G	1.054 (0.952-1.166)	5.79E-01	3.12E-01
9	rs4077515	139266496	31.6%	30.0%	T	1.062 (0.974-1.158)	1.76E-01	1.71E-01
16	rs11150612	31357760	69.9%	67.1%	A	1.200 (1.099-1.311)	8.65E-05	4.89E-05
8	rs10086568	6900336	32.1%	28.4%	A	1.195 (1.096-1.303)	1.72E-05	4.98E-05

Supplementary table 3. Look-up of previously-reported SNPs in the current GWAS discovery sample of 1,434 cases and 10,661 controls

Imputed HLA allele	Imputation r^2	Freq cases	Freq controls	OR (95% CI)	P
			**Yu et al[2]**		
HLA-B*4001	0.97	18.89%	16.78%	1.301 (1.173-1.443)	6.34E-07
HLA-DQB1*0302	0.91	8.76%	7.15%	1.441 (1.248-1.664)	6.44E-07
HLA-A*1101	0.98	31.46%	27.10%	1.209 (1.108-1.320)	2.02E-05
HLA-DRB1*0901	0.97	12.59%	16.11%	0.746 (0.662-0.840)	1.29E-06
HLA-DQB1*0303	0.94	12.66%	16.38%	0.736 (0.653-0.828)	3.98E-07
HLA-DQA1*0102	0.97	14.98%	17.40%	0.761 (0.681-0.851)	1.75E-06
HLA-DQB1*0201	0.94	7.65%	10.93%	0.693 (0.598-0.803)	1.01E-06
			**Gharavi et al[4]**		
HLA-DQB1*0602	0.95	2.86%	4.85%	0.605 (0.479-0.763)	2.16E-05
HLA-DRB1*1501	0.93	7.90%	9.52%	0.730 (0.630-0.846)	2.91E-05
			**Kiryluk et al[5]**		
HLA-DQA1*0101	0.97	14.71%	10.33%	1.299 (1.157-1.458)	8.95E-06
HLA-DQA1*0102	0.97	14.98%	17.40%	0.761 (0.681-0.851)	1.75E-06
HLA-DQB1*0201	0.94	7.65%	10.93%	0.693 (0.598-0.803)	1.01E-06
HLA-DQB1*0301	0.91	28.71%	24.80%	1.191 (1.089-1.301)	1.25E-04
			**This study**		
HLA-DRB1*04	0.97	16.93%	13.67%	1.446 (1.297-1.613)	3.16E-11
HLA-DRB1*0403	0.63	2.96%	1.94%	1.754 (1.379-2.232)	4.69E-06
HLA-DRB1*0405	0.96	8.07%	6.72%	1.292 (1.114-1.498)	7.19E-04
HLA-DRB1*0404	0.67	1.85%	1.46%	1.478 (1.100-1.984)	9.42E-03
HLA-DRB1*0406	0.88	3.35%	3.29%	1.179 (0.945-1.471)	1.45E-01
HLA-DRB1*0401	0.68	0.66%	0.69%	0.990 (0.621-1.579)	9.67E-01
HLA-DRB1*0410	0.11	0.00%	0.11%	-	9.97E-01
HLA-DPB1*02	0.91	27.95%	23.17%	1.318 (1.205-1.442)	1.77E-09
HLA-DPB1*0202	0.89	9.39%	7.11%	1.328 (1.155-1.527)	6.63E-05
HLA-DPB1*0201	0.87	18.52%	16.15%	1.229 (1.109-1.363)	9.12E-05

Supplementary table 4. Previously reported and newly-identified HLA alleles associated with IgAN in discovery samples (Stage 1), imputed with the SNP2HLA tool[6].

SNP	Chr:position	Risk/non-risk allele	Locus	Northern		Southern		Combined GWAS + All Validation		
				OR	P	OR	P	P	OR	Phet
rs2738058	chr8:6821617	T/C	*DEFA8P/DEFA1*	1.245	7.08E-05	1.327	1.92E-06	4.28E-21	1.255	0.2596
rs7190997	chr16:31368178	C/T	*ITGAX*	1.064	3.01E-01	1.129	3.91E-02	2.01E-16	1.226	0.0009
rs2074038	chr11:44087989	T/G	*ACCS*	1.054	3.24E-01	1.177	4.08E-03	1.47E-08	1.145	0.2564
rs2033562	chr8:103547739	C/G	*x*	1.139	9.15E-03	1.074	1.77E-01	3.09E-08	1.130	0.5769
rs7634389	chr3:186738421	C/T	*ST6GAL1*	1.176	1.48E-03	1.075	1.80E-01	1.01E-07	1.126	0.5733
rs9314614	chr8:6697731	C/G	*LOC100652791*	1.051	3.42E-01	1.135	2.10E-02	1.07E-07	1.130	0.3186
rs11264799	chr1: 157670757	C/T	*FCRL3*	1.086	1.82E-01	1.015	8.19E-01	1.78E-07	1.152	0.0571
rs7979707	chr12:54836257	C/T	*GTSF1/ITGA5*	0.897	5.34E-01	1.233	8.15E-02	2.30E-07	1.313	0.0731
rs12581651	chr12:7162598	A/G	*x*	1.242	1.20E-01	1.209	1.39E-01	1.70E-06	1.295	0.762
rs137975261	chr16:86556644	A/G	*x*	1.920	3.72E-01	1.743	3.90E-01	1.80E-05	2.111	0.7546
rs7305528	chr12:70852590	A/G	*x*	1.004	9.44E-01	0.978	6.92E-01	2.34E-05	1.110	0.0072
rs75131344	chr2:61935818	T/C	*x*	1.069	3.77E-01	1.119	2.04E-01	2.69E-05	1.165	0.1891
rs6773807	chr3:20220273	C/T	*SGOL1*	0.949	7.27E-01	1.168	2.77E-01	1.27E-04	1.248	0.0615
rs34104149	chr1:209318792	G/T	*LOC642587*	0.904	2.59E-01	1.248	3.45E-02	1.70E-04	1.180	0.002
rs17145884	chr11:62200176	T/C	*AHNAK, ASRGL1*	0.980	7.05E-01	0.986	8.15E-01	2.31E-04	1.095	0.0013
rs10899844	chr10:44118060	T/C	*x*	0.996	9.46E-01	1.096	1.26E-01	2.39E-04	1.094	0.0778
rs11764463	chr7:105875903	T/C	*NAMPT*	1.041	6.06E-01	1.048	5.30E-01	2.58E-04	1.123	0.052
rs12868611	chr13:32444439	C/T	*EEF1DP3*	0.972	5.96E-01	1.041	4.73E-01	5.26E-04	1.085	0.0237
rs13133504	chr4:176252966	C/T	*x*	1.089	3.13E-01	0.946	6.16E-01	7.20E-04	1.165	0.0349
rs3804780	chr3:4352981	G/A	*SETMAR*	0.966	7.08E-01	1.055	5.82E-01	1.37E-03	1.140	0.0238
rs6435051	chr2:201329213	A/G	*SPATS2L*	0.986	8.09E-01	0.877	2.59E-02	4.18E-03	1.074	<0.001
rs2421790	chr1:71191807	A/G	*x*	0.852	3.82E-03	1.007	9.06E-01	9.87E-03	1.066	<0.001

Supplementary table 5. 22 SNPs analysed in validation 2 samples.

Risk alleles were defined based on direction of effect at the discovery (GWAS) stage.

SNP/locus I^2/P_{het}	Risk/non-risk allele	Sample	Risk allele frequency Cases	Risk allele frequency Controls	P	OR(95% CI)	I2	P_{het}
**rs11264799**	C/T	GWAS logistic	81.0%	77.5%	1.43E-05	1.239(1.125-1.365)		
		GWAS gemma			1.77E-05			
**chr1:157670757**		Validation 1	80.4%	77.5%	1.73E-04	1.194(1.088-1.309)		
FCRL3		Validation 2 Southern	77.9%	77.6%	8.19E-01	1.015(0.894-1.152)		
		Validation 2 Northern	79.2%	77.8%	1.82E-01	1.086(0.962-1.226)		
		Validation 3 Southern	78.9%	77.2%	1.75E-01	1.104(0.957-1.273)		
		Validation 3 Northern	79.1%	78.9%	8.94E-01	1.012(0.849-1.206)		
		All validation			4.42E-04	1.104 (1.045-1.166)		
		Meta-analysis			2.00E-07	1.135(1.082-1.191)	50.4	0.090

Supplementary Table 6. Association results at the suggestive locus 1q23.1

a) Imputation info scores by IMPUTEv2 (info) and minor allele frequencies (maf) in cases and controls, separated by genotyping platform (Illumina array).

		660-Quad (Cases)	OmniExpress (Controls)	1M-Duo (Controls)	550K (Controls)	610-Quad (Controls)
**average info across all SNPs**		0.36	0.33	0.33	0.31	0.31
**% info > 0.8**		32.7%	31.8%	32.1%	30.5%	30.7%
**rs2074038**	**info**	0.99	0.98	0.98	0.99	0.99
	**maf**	32.2%	28.9%	26.7%	28.5%	28.8%
**rs7190997**	**info**	0.95	typed	typed	0.94	0.97
	**maf**	27.3%	31.8%	30.7%	29.4%	30.1%
**rs7634389**	**info**	0.98	0.98	0.98	0.99	0.98
	**maf**	47.4%	43.8%	45.3%	42.1%	42.9%
**rs9314614**	**info**	0.99	0.99	0.99	0.99	0.99
	**maf**	39.7%	35.5%	35.3%	37.0%	36.1%
**rs2738058**	**info**	typed	0.99	typed	typed	typed
	**maf**	27.0%	33.9%	34.1%	33.0%	33.2%
**rs12716641**	**info**	0.99	0.99	0.99	0.99	0.99
	**maf**	21.7%	26.7%	25.1%	25.9%	25.2%
**rs2033562**	**info**	1.00	1.00	1.00	0.99	1.00
	**maf**	51.4%	48.4%	46.8%	46.6%	47.8%

b) Association results in the full expanded GWAS dataset compared to the subset of samples from the previous GWAS (adjusted for principal components previously calculated)[2].

SNP/locus Risk/non-risk allele	SNP type	Risk allele frequency Cases	Expanded GWAS (1434 cases, 10,661 controls)			Previous GWAS[2] (1434 cases, 4270 controls)		
			Risk allele frequency Controls	OR (95% CI)	P	Risk allele frequency Controls	OR (95% CI)	P
rs2738058 8:6821617 *DEFA8P* T/C	genotyped	73.0%	66.6%	1.310 (1.203-1.427)	5.75E-10	67.4%	1.289 (1.173-1.415)	1.16E-07
rs7190997 16: 31368178 *ITGAX-ITGAM* C/T	imputed	72.0%	69.2%	1.207 (1.103-1.320)	4.00E-05	70.0%	1.219 (1.106-1.343)	6.79E-05
rs2074038 11:44087989 *ACCS* T/G	imputed	32.3%	28.8%	1.207 (1.104-1.319)	3.36E-05	28.1%	1.248 (1.134-1.375)	6.31E-06
rs2033562 8:103547739 *KLF10/ODF1* C/G	imputed	51.4%	47.7%	1.176 (1.085-1.274)	7.98E-05	47.7%	1.161 (1.065-1.266)	7.18E-04
rs7634389 3:186738421 *ST6GAL1* C/T	imputed	47.4%	43.5%	1.151 (1.061-1.248)	6.80E-04	43.5%	1.164 (1.066-1.270)	7.17E-04
rs9314614 8:6697731 *XKR5* C/G	imputed	39.7%	36.0%	1.191 (1.097-1.295)	3.54E-05	36.2%	1.166 (1.067-1.275)	7.24E-04
rs12716641 8:6898998 *DEFA7P* T/C	imputed	78.0%	74.3%	1.260 (1.150-1.381)	7.80E-07	75.2%	1.227 (1.109-1.356)	6.76E-05

Supplementary table 7. Exclusion of batch effects influencing results at the top 7 SNPs

a) Discovery GWAS (Stage 1) results from separate analyses of discovery GWAS samples in two clusters of Northern and Southern Chinese samples (Supplementary figure 3) with adjustment of the first five principal components in each stage. These are compared with results from the original analysis combining Northern and Southern Chinese samples with adjustment of the first five principal components.

**SNP**		**Risk allele**	**Northern cluster, 414 cases, 2306 controls**			**Southern cluster, 1020 cases, 8355 controls**		
			**F_A**	**F_U**	**OR (95% CI)**	**F_A**	**F_U**	**OR (95% CI)**
rs7634389	*ST6GAL1*	C	47.0%	44.7%	1.088 (0.922-1.283)	47.6%	42.8%	1.173 (1.067-1.291)
rs9314614	*XKR5*	C	40.3%	37.4%	1.151 (0.978-1.355)	39.5%	35.6%	1.201 (1.088-1.326)
rs2738058	*DEFA8P*	T	73.6%	67.4%	1.331 (1.115-1.588)	72.8%	66.4%	1.310 (1.185-1.448)
rs12716641	*DEFA7P*	T	80.0%	77.0%	1.241 (1.024-1.505)	77.7%	73.8%	1.257 (1.130-1.398)
rs2033562	*KLF10/ODF1*	C	52.8%	47.7%	1.238 (1.054-1.454)	50.8%	47.7%	1.160 (1.055-1.275)
rs2074038	*ACCS*	T	34.0%	33.0%	1.115 (0.943-1.318)	31.5%	27.4%	1.248 (1.122-1.389)
rs7190997	*ITGAX*	C	77.8%	74.6%	1.337 (1.109-1.611)	70.8%	68.2%	1.169 (1.053-1.297)

**SNP**	**Locus**	**Risk allele**	**Meta-analysis of North and South**				**Original analysis (combined)**	
			**OR**	$P_{logistic}$ λ=1.079	P_{het}	I^2	$P_{logistic}$ λ=1.087	P_{gemma} λ=1.039
rs7634389	*ST6GAL1*	C	1.151	8.15E-04	0.437	0	6.80E-04	4.33E-05
rs9314614	*XKR5*	C	1.187	6.99E-05	0.666	0	3.54E-05	6.20E-05
rs2738058	*DEFA8P*	T	1.315	8.04E-10	0.879	0	5.75E-10	1.51E-10
rs12716641	*DEFA7P*	T	1.253	2.08E-06	0.912	0	7.80E-07	1.79E-06
rs2033562	*KLF10/ODF1*	C	1.179	7.68E-05	0.494	0	7.98E-05	4.14E-04
rs2074038	*ACCS*	T	1.208	3.66E-05	0.264	19.98	3.36E-05	4.45E-05
rs7190997	*ITGAX*	C	1.207	5.35E-05	0.219	33.85	4.00E-05	1.94E-05

b) Meta-analysis across stages 1-4 for all Northern (2,581 cases, 4,794 controls) and Southern (5,732 cases, 14,886 controls) samples.

SNP/locus	Risk/non-risk allele	Sample	Risk allele frequency Cases	Risk allele frequency Controls	P	OR (95% CI)	P_{het}	P_{het} North vs South
**rs7634389**	**C/T**	GWAS Southern	47.6%	42.8%	1.01E-03	1.173 (1.067-1.291)		
		Validation 1	46.2%	43.7%	8.86E-03	1.105 (1.026-1.191)		
		Validation 2 Southern	46.1%	44.3%	1.80E-01	1.075 (0.968-1.194)		
**3:186738421**		Validation 3 Southern	47.0%	42.8%	5.94E-03	1.180 (1.049-1.328)		
ST6GAL1		All Southern			6.52E-07	1.127 (1.075-1.181)	0.510	
		GWAS Northern	47.0%	44.7%	3.18E-01	1.088 (0.922-1.283)		
		Validation 2 Northern	46.6%	42.6%	1.48E-03	1.176 (1.064-1.300)		
		Validation 3 Northern	46.7%	43.8%	1.09E-01	1.124 (0.974-1.297)		
		All Northern			3.16E-04	1.144 (1.063-1.232)	0.702	
		Combined			8.65E-10	1.132 (1.088-1.178)	0.791	0.729
**rs9314614**	**C/G**	GWAS Southern	39.5%	35.6%	2.90E-04	1.201 (1.088-1.326)		
		Validation 1	37.7%	35.0%	3.77E-03	1.121 (1.038-1.211)		
		Validation 2 Southern	38.6%	35.6%	2.10E-02	1.135 (1.019-1.264)		
**8:6697731**		Validation 3 Southern	38.4%	36.0%	1.03E-01	1.108 (0.980-1.253)		
XKR5		All Southern			1.14E-07	1.141 (1.086-1.197)	0.693	
		GWAS Northern	40.3%	37.4%	9.05E-02	1.151 (0.978-1.355)		
		Validation 2 Northern	40.2%	39.1%	3.42E-01	1.051 (0.948-1.165)		
		Validation 3 Northern	41.9%	36.8%	4.85E-03	1.234 (1.066-1.428)		
		All Northern			3.66E-03	1.117 (1.037-1.204)	0.196	
		Combined			1.63E-09	1.134 (1.088-1.181)	0.554	0.651
**rs2738058**	**T/C**	GWAS Southern	72.8%	66.4%	1.37E-07	1.310 (1.185-1.448)		
		Validation 1	72.4%	69.0%	6.10E-05	1.181 (1.089-1.281)		
		Validation 2 Southern	71.9%	66.0%	1.92E-06	1.327 (1.181-1.491)		
**8:6821617**		Validation 3 Southern	72.6%	67.3%	8.74E-05	1.303 (1.142-1.487)		
DEFA8P		All Southern			1.13E-18	1.260 (1.197-1.326)	0.261	
		GWAS Northern	73.6%	67.4%	1.55E-03	1.331 (1.115-1.588)		
		Validation 2 Northern	72.3%	67.6%	7.08E-05	1.245 (1.117-1.386)		

		Validation 3 Northern	73.4%	67.0%	1.64E-04	1.354 (1.156-1.585)		
		All Northern			4.04E-10	1.289 (1.190-1.396)	0.637	
		Combined			3.38E-27	1.268 (1.215-1.324)	0.526	0.633
**rs12716641**	**T/C**	GWAS Southern	77.7%	73.8%	2.61E-05	1.257 (1.130-1.398)		
		Validation 1	78.2%	73.6%	1.22E-08	1.287 (1.180-1.404)		
		Validation 2 Southern	78.8%	75.2%	1.81E-03	1.219 (1.076-1.381)		
**8:6898998**		Validation 3 Southern	78.7%	75.5%	1.00E-02	1.204 (1.045-1.386)		
DEFA7P		All Southern			5.73E-16	1.253 (1.187-1.324)	0.835	
		GWAS Northern	80.0%	77.0%	2.77E-02	1.241 (1.024-1.505)		
		Validation 2 Northern	80.4%	78.4%	5.44E-02	1.129 (0.998-1.278)		
		Validation 3 Northern	80.5%	77.4%	3.85E-02	1.207 (1.010-1.442)		
		All Northern			5.23E-04	1.173 (1.072-1.283)	0.673	
		Combined			2.80E-18	1.231 (1.175-1.290)	0.785	0.216
**rs2033562**	**C/G**	GWAS Southern	50.8%	47.7%	2.23E-03	1.160 (1.055-1.275)		
		Validation 1	50.5%	47.7%	3.45E-03	1.116 (1.037-1.201)		
		Validation 2 Southern	50.6%	48.8%	1.77E-01	1.074 (0.968-1.192)		
**8:103547739**		Validation 3 Southern	49.8%	49.0%	5.90E-01	1.033 (0.918-1.163)		
KLF10/ODF1		All Southern			3.12E-05	1.104 (1.054-1.157)	0.458	
		GWAS Northern	52.8%	47.7%	9.34E-03	1.238 (1.054-1.454)		
		Validation 2 Northern	50.8%	47.4%	9.15E-03	1.139 (1.033-1.256)		
		Validation 3 Northern	50.9%	45.0%	1.35E-03	1.265 (1.096-1.460)		
		All Northern			2.57E-06	1.190 (1.107-1.279)	0.429	
		Combined			1.45E-09	1.129 (1.085-1.174)	0.308	0.091
**rs2074038**	**T/G**	GWAS Southern	31.5%	27.4%	4.44E-05	1.248 (1.122-1.389)		
		Validation 1	31.3%	28.6%	1.87E-03	1.137 (1.049-1.233)		
		Validation 2 Southern	32.5%	28.9%	4.08E-03	1.177 (1.053-1.315)		
**11:44087989**		Validation 3 Southern	30.4%	29.1%	3.42E-01	1.066 (0.935-1.216)		
ACCS		All Southern			1.69E-08	1.159 (1.101-1.220)	0.294	
		GWAS Northern	34.0%	33.0%	2.05E-01	1.115 (0.943-1.318)		

		Validation 2 Northern	34.8%	33.6%	3.24E-01	1.054 (0.950-1.170)		
		Validation 3 Northern	35.6%	32.7%	1.03E-01	1.134 (0.975-1.318)		
		All Northern			3.30E-02	1.086 (1.007-1.173)	0.695	
		Combined			4.35E-09	1.136 (1.089-1.185)	0.385	0.167
**rs7190997**	**C/T**	GWAS Southern	70.8%	68.2%	3.41E-03	1.169 (1.053-1.297)		
		Validation 1	73.5%	66.7%	3.35E-15	1.386 (1.278-1.503)		
		Validation 2 Southern	71.9%	69.3%	3.91E-02	1.129 (1.006-1.267)		
**16: 31368178**		Validation 3 Southern	72.9%	68.4%	1.16E-03	1.239 (1.089-1.411)		
ITGAX-ITGAM		All Southern			6.24E-18	1.254 (1.191-1.320)	0.013	
		GWAS Northern	77.8%	74.6%	2.35E-03	1.337 (1.109-1.611)		
		Validation 2 Northern	77.3%	76.2%	3.01E-01	1.064 (0.946-1.196)		
		Validation 3 Northern	77.5%	74.7%	7.30E-02	1.166 (0.986-1.379)		
		All Northern			2.23E-03	1.143 1.049-1.245)	0.123	
		Combined			2.98E-19	1.223 (1.171-1.279)	0.005	0.067

Supplementary table 8. Comparisons of association results in Northern and Southern Chinese samples

rsId	Locus	OR	Stage 1 P		Stage 1 SE		Meta-analysis	
			original	adjusted	original	adjusted	original	adjusted
rs7634389	*ST6GAL1*	1.151	6.80E-04	1.12E-03	0.041	0.043	7.27E-10	1.16E-09
rs9314614	*XKR5*	1.191	3.54E-05	7.29E-05	0.042	0.044	9.48E-10	1.81E-09
rs2738058	*DEFA8P*	1.310	5.75E-10	2.78E-09	0.044	0.045	2.31E-27	1.06E-26
rs12716641	*DEFA7P*	1.260	7.80E-07	2.15E-06	0.047	0.049	1.13E-18	3.02E-18
rs2033562	*KLF10/ODF1*	1.176	7.98E-05	1.54E-04	0.041	0.043	1.41E-09	2.57E-09
rs2074038	*ACCS*	1.207	3.36E-05	6.94E-05	0.045	0.047	3.93E-09	7.39E-09
rs7190997	*ITGAX*	1.207	4.00E-05	8.15E-05	0.046	0.048	2.26E-19	4.46E-19

Supplementary table 9. Impact of adjusting for lambda GC (lambda=1.087) at the discovery stage (Stage 1) and in the meta-analysis across all four stages.

a) Association results with and without adjusting for age and gender in the meta-analysis across all four stages, in the subset of samples (6,323 cases, 17,349 controls; 84.6%) for which this information is available.

Locus	CHR	SNP	BP	Risk/non-risk allele	Without adjustment for age and gender		With adjustment for age and gender	
					**P**	**OR (95% CI)**	**P**	**OR (95% CI)**
ST6GAL1	3	rs7634389	186738421	C/T	1.27E-09	1.145 (1.096-1.196)	3.85E-09	1.143 (1.093-1.195)
XKR5	8	rs9314614	6697731	C/G	7.13E-10	1.151 (1.101-1.204)	6.61E-09	1.145 (1.094-1.198)
DEFA8P	8	rs2738058	6821617	T/C	6.27E-22	1.266 (1.207-1.329)	1.17E-22	1.278 (1.217-1.342)
DEFA7P	8	rs12716641	6898998	T/C	1.34E-15	1.239 (1.176-1.306)	7.80E-16	1.247 (1.182-1.316)
KLF10/ODF1	8	rs2033562	103547739	C/G	4.26E-09	1.139 (1.091-1.190)	2.65E-08	1.134 (1.085-1.185)
ACCS	11	rs2074038	44087989	T/G	6.33E-07	1.126 (1.075-1.180)	1.13E-06	1.126 (1.073-1.181)
ITGAX	16	rs7190997	31368178	C/T	1.29E-12	1.194 (1.137-1.254)	4.58E-12	1.193 (1.135-1.254)

b) Stratified analysis in males and females, among 6,391 cases and 17,364 controls with gender information available. Association results are based on the meta-analysis across all four stages.

Locus	CHR	SNP	BP	Males (3,067 cases, 9,993 controls)		Females (3,324 cases, 7,371 controls)		P_{het} males/ females
				**P**	**OR (95% CI)**	**P**	**OR (95% CI)**	
ST6GAL1	3	rs7634389	186738421	3.87E-05	1.137 (1.069-1.208)	1.07E-05	1.152 (1.082-1.227)	0.766
XKR5	8	rs9314614	6697731	1.94E-05	1.146 (1.077-1.220)	5.07E-06	1.162 (1.089-1.239)	0.764
DEFA8P	8	rs2738058	6821617	2.65E-12	1.274 (1.190-1.363)	2.57E-11	1.262 (1.178-1.351)	0.844
DEFA7P	8	rs12716641	6898998	1.55E-07	1.219 (1.132-1.312)	3.61E-09	1.253 (1.163-1.350)	0.606
KLF10/ODF1	8	rs2033562	103547739	1.74E-05	1.142 (1.075-1.213)	1.88E-04	1.126 (1.058-1.199)	0.755
ACCS	11	rs2074038	44087989	4.67E-03	1.099 1.029-1.173)	3.79E-05	1.152 (1.077-1.233)	0.321
ITGAX	16	rs7190997	31368178	5.74E-07	1.189 (1.111-1.273)	2.93E-06	1.184 (1.103-1.271)	0.932

Supplementary table 10. Impact of adjusting for age and gender on the association results

SNP/locus	Risk/non-risk allele	Sample		Risk allele frequency Cases	Risk allele frequency Controls	P	OR(95% CI)
rs6677604	G/A	GWAS	Combined	95.0%	94.4%	2.60E-01	1.107 (0.927-1.322)
1:196686918			Northern	94.8%	93.1%	1.40E-01	1.310 (0.915-1.874)
CFH			Southern	95.0%	94.8%	7.17E-01	1.041 (0.836-1.296)
		Validation 2	Northern	94.3%	92.6%	7.34E-03	1.327 (1.079-1.633)
			Southern	95.3%	94.4%	1.24E-01	1.212 (0.949-1.548)
		Validation 3	Northern	92.8%	91.9%	3.70E-01	1.130 (0.865-1.478)
			Southern	95.3%	94.7%	3.34E-01	1.146 (0.869-1.513)
		Combined	Northern			2.35E-03	1.260 (1.086-1.463)
			Southern			1.08E-01	1.122 (0.975-1.292)
P_{het}/I^2	0.70/0		All			1.12E-03	1.185 (1.070-1.313)

Supplementary table 11. Association at the *CFH* locus in Stage 1, 3 and 4 samples, comparing effects in Northern and Southern Chinese samples.

**Haplotype** rs9314614-rs2738058-rs12716641	**# risk alleles**	**P**	**Average frequency (controls)**	**OR (each vs all)**	**Phet**	**OR with CTT as reference**
CTT	3	4.71E-22	24.6%	1.257	0.365	1.000
CTC	2	7.88E-01	4.0%	0.987	0.914	0.850
CCT	2	1.95E-01	3.7%	0.934	0.284	0.792
GTT	2	1.34E-03	34.2%	1.068	0.738	0.896
CCC	1	4.20E-05	3.9%	0.809	0.689	0.709
GTC	1	2.31E-02	4.5%	0.898	0.004	0.767
GCT	1	9.74E-08	13.4%	0.847	0.060	0.744
GCC	0	3.51E-13	11.5%	0.777	0.643	0.688

Supplementary table 12. Haplotype analysis of three independent SNPs identified at the *DEFA* locus, showing the presence of eight different haplotypes at frequencies expected at linkage equilibrium, and a general trend/additive effect between the number of risk alleles carried by each haplotype and the odds ratio of that haplotype.

SNP/Locus	Physical location	eQTL/ENCODE	Other diseases	Known functions of genes
rs7190997<br>16p11.2	• Lies within an intron of the *ITGAX* gene<br>• In LD with a missense SNP in *ITGAX* rs2230429 p.Pro517Arg (r^2=0.84 in 1000 genomes Asians)[10] and the previously reported rs[1]1150612 (r^2=0.88 in our samples) [5]	• Risk allele is strongly associated with increased expression levels of the *ITGAX* gene ($P<9.81x10^{-198}$) in peripheral blood cells, monocytes (rs7206295, r^2=0.82, $P=7.75x10^{-9}$) and also of the *ITGAM* gene in peripheral blood cells ($P=1.36x10^{-24}$) and monocytes ($P=6.51x10^{-7}$).<br>• In LD (r^2>0.8) with SNPs predicted to affect transcription factor binding and chromatin structure.	• Variants at this locus have previously been associated with systemic lupus erythematosus (SLE) in European populations[13-14]<br>• Reported SLE risk alleles and those in LD with these SNPs in HapMap Europeans are either very rare (MAF <1%) or absent in both our samples and HapMap Asians.<br>• Risk alleles for SLE are generally protective against IgAN.[5,15,16]	• *ITGAX* encodes the integrin alpha X chain protein, which combines with the beta 2 chain (encoded by *ITGB2*) to form a leukocyte-specific integrin hetero dimer complex referred to as inactivated-C3b receptor 4 (CR4).<br>• This complex is known to play a role in the adherence of neutrophils and monocytes to stimulated endothelium cells and in the phagocytosis of complement-coated particles.<br>• *ITGAM* is known to regulate IgA-producing cells in the intestines of mice.[12]
rs9314614<br>8p23	• Located in the intron of the long-coding RNA *GS1-24F4.2.* 30kb away from *DEFB1 and adjacent to XKR5* X Kell Blood Group Precursor-Related Family	• Risk allele is associated with expression of the gene *DEFB1* (tagged by rs2977798; r^2>0.8) in monocytes ($P=4.22x10^{-4}$)<br>• May affect chromatin structure, binding of CTCF and other proteins	• Not previously associated with any other trait.	• *DEFB1* involved in immune response<br>• Member of the defensin family, microbicidal and cytotoxic peptides produced by neutrophils<br>• Antimicrobial peptide, may be involved in resistance of epithelial surfaces to microbial colonization

Supplementary table 13. Functional annotations of recently reported loci and associated SNPs (details on eQTLs and ENCODE annotations in Supplementary tables 14-15, Supplementary data 2 and 3)[5,7-16]

chromosome	Position	SNP id	R2[%]	Candidate gene*	Meta z score^	Meta p value #	Alleles	Allele assessed
11	44085687	rs7127924	0.995501	*ACCS*	31.79866	9.81E-198	C/A	A
11	44087989	rs2074038	1	*ACCS*	42.79569	9.81E-198	G/T	T
11	44091399	rs7951555	0.891488	*ACCS*	35.36956	9.81E-198	A/G	G
11	44093323	rs2074040	0.867031	*ACCS*	35.4295	9.81E-198	C/G	G
16	31336519	rs7206295	0.832116	*ITGAX*	-40.2033	9.81E-198	C/T	T
16	31340909	rs4077810	0.86951	*ITGAX*	-39.6776	9.81E-198	C/T	T
16	31343653	rs3087796	0.87255	*ITGAX*	-40.5388	9.81E-198	G/A	A
16	31343769	rs4597342	0.872532	*ITGAX*	-40.5394	9.81E-198	C/T	T
16	31347557	rs9673519	0.887398	*ITGAX*	-40.5783	9.81E-198	C/T	T
16	31348233	rs4075052	0.887373	*ITGAX*	-40.586	9.81E-198	C/A	A
16	31348848	rs4608351	0.88758	*ITGAX*	-40.602	9.81E-198	C/T	T
16	31355657	rs11150611	0.895089	*ITGAX*	-40.6253	9.81E-198	G/C	C
16	31357810	rs11150613	0.896697	*ITGAX*	-40.1915	9.81E-198	T/C	C
16	31361643	rs4459557	0.912921	*ITGAX*	-40.8901	9.81E-198	C/G	G
16	31363977	rs11574631	0.877172	*ITGAX*	-42.0613	9.81E-198	T/C	C
16	31366016	rs11150614	0.979853	*ITGAX*	-42.1303	9.81E-198	G/A	A
16	31368178	rs7190997	1	*ITGAX*	30.95974	9.81E-198	C/T	C
16	31360944	rs12923297	0.887421	*ITGAX*	-28.0678	2.42E-173	T/C	T
16	31347748	rs3925075	0.863108	*ITGAX*	-27.9664	4.17E-172	G/A	A
16	31347350	rs4506917	0.863273	*ITGAX*	-27.9379	9.27E-172	T/G	T
16	31359826	rs11645917	0.891087	*ITGAX*	-27.7002	6.94E-169	T/C	C
16	31334236	rs11150610	0.800358	*ITGAX*	23.83516	1.45E-125	A/C	A
16	31336719	rs1143682	0.853171	*ITGAX*	22.71235	3.39E-114	G/A	A
16	31363788	rs11574630	0.892581	*ITGAX*	22.27453	6.54E-110	T/C	T
16	31362263	rs4889649	0.894909	*ITGAX*	21.98276	4.23E-107	T/G	G
16	31363417	rs10782004	0.891273	*ITGAX*	21.73203	1.02E-104	G/A	A
16	31357760	rs11150612	0.876378	*ITGAX*	21.57095	3.38E-103	G/A	A
11	44091399	rs7951555	0.891488	*EXT2*	15.99954	1.29E-57	A/G	G
11	44093323	rs2074040	0.867031	*EXT2*	15.92642	4.16E-57	C/G	G
11	44087989	rs2074038	1	*EXT2*	15.36924	2.63E-53	G/T	T
11	44085687	rs7127924	0.995501	*EXT2*	13.40393	5.74E-41	C/A	A
11	44093323	rs2074040	0.867031	*EXT2*	10.41335	2.16E-25	C/G	G

11	44091399	rs7951555	0.891488	*EXT2*	10.37437	3.25E-25	A/G	G
11	44087989	rs2074038	1	*EXT2*	10.12433	4.31E-24	G/T	T
11	44085687	rs7127924	0.995501	*EXT2*	9.146848	5.86E-20	C/A	A
3	186732725	rs3821819	0.949871	*ST6GAL1*	-9.14501	5.96E-20	G/A	A
16	31368178	rs7190997	1	*AC093520.4,ITGAM*	10.24	1.36E-24	C/T	C
16	31368178	rs7190997	1	*BCKDK*	9.93	3.17E-23	C/T	C
3	186734466	rs967367	0.950775	*ST6GAL1*	-9.13104	6.79E-20	G/A	A
3	186732679	rs17776120	0.950398	*ST6GAL1*	-9.10268	8.82E-20	C/A	A

Supplementary table 14: Association between genotypes and mRNA expression levels in whole blood using an eQTL meta-analysis (data taken from Blood eQTL browser http://genenetwork.nl/bloodeqtlbrowser/; Westra et. al. 2013[7])

R2$^{\%}$ is the r^2 value between the SNP and the reported risk SNP within the locus
***Candidate gene** is the gene whose expression is affected as determined in the whole blood based eQTL meta-analysis
^ **Meta z score** and $^{\#}$**Meta p value** is the combined z score and p value obtained in the eQTL meta-analysis when analyzing all the datasets together using data from Westra et al (2013). A positive z score (greater than 0) implies an increase in expression for the allele assessed, while a negative z score implies the allele assessed associates to a reduction in gene expression in comparison with the other allele.

				Monocytes				B-cells			
**SNP_B**	**CHR_B**	**BP_B**	**R2**	**p.spearman**	**P**	**BETA**	**Gene**	**p.spearman**	**P**	**BETA**	**Gene**
rs2074040	11	44093323	0.867031	4.96E-24	1.07E-36	0.7947	*ACCS*	2.33E-23	1.46E-29	0.638	*ACCS*
rs2074040	11	44093323	0.867031	2.45E-22	1.94E-35	0.7603	*PHACS*	8.06E-19	2.32E-22	0.5992	*PHACS*
rs7951555	11	44091399	0.891488	2.45E-22	1.94E-35	0.7603	*PHACS*	8.06E-19	2.32E-22	0.5992	*PHACS*
rs4075052	16	31348233	0.887373	1.94E-11	7.71E-14	-0.1612	*ITGAM*	-	-	-	-
rs4597342	16	31343769	0.872532	1.94E-11	7.71E-14	-0.1612	*ITGAM*	-	-	-	-
rs11150614	16	31366016	0.979853	3.64E-11	1.89E-12	-0.1562	*ITGAM*	-	-	-	-
rs7206295	16	31336519	0.832116	1.56E-10	1.01E-12	-0.1547	*ITGAM*	-	-	-	-
rs7206295	16	31336519	0.832116	2.31E-08	7.75E-09	-0.1456	*ITGAX*	-	-	-	-
rs8060268	16	31345280	0.881129	1.59E-10	1.07E-12	-0.1546	*ITGAM*	-	-	-	-
rs8060268	16	31345280	0.881129	9.24E-08	2.03E-08	-0.1417	*ITGAX*	-	-	-	-
rs7190997	16	31368178	1	3.01E-06	6.51E-07	-0.1032	*ITGAM*	-	-	-	-
rs3925075	16	31347748	0.863108	3.17E-06	2.44E-07	-0.1067	*ITGAM*	-	-	-	-
rs3925075	16	31347748	0.863108	5.11E-05	9.25E-05	-0.09343	*ITGAX*	-	-	-	-
rs2977798	8	6697450	0.999287	0.000165	0.000422	0.09989	*DEFB1*	-	-	-	-
rs17776120	3	186732679	0.950398	-	-	-		3.6E-07	1.61E-07	-0.2581	*ST6GAL1*

Supplementary table 15: Association between genotypes and mRNA expression levels in monocytes and B-cells using an eQTL analysis (data taken from Fairfax et. al. 2012)[8]

Locus	SNP	Risk allele	YRI	CEU	CHB+JPT
Gharavi et al[3]					
CFH	**rs3766404**	**T**	**0.45**	**0.85**	**0.92**
CFH	**rs6677604**	**G**	**0.51**	**0.76**	**0.92**
HLA	rs2856717	G	0.63	0.63	0.79
HLA	rs9275596	T	0.70	0.59	0.83
HLA	rs9357155	G	0.93	0.85	0.85
HLA	rs2071543	G	0.92	0.83	0.84
HLA	rs3129269	C	0.83	0.67	0.79
HLA	rs1883414	G	0.89	0.68	0.81
MTMR3/HORMAD2	**rs2412971**	**G**	**0.22**	**0.57**	**0.65**
MTMR3/HORMAD2	**rs2412973**	**C**	**0.22**	**0.62**	**0.68**
Yu et al[1]					
DEFA	rs2738048	T	0.82	0.71	0.64
TNFSF13	rs3803800	A	0.78	0.22	0.30
TNFSF13	rs4227	G	0.54	0.25	0.18
MTMR3/HORMAD2	**rs12537**	C	**0.53**	**0.69**	**0.80**
HLA-A	rs2523946	C	0.51	0.52	0.51
HLA-DRB1	rs660895	G	0.10	0.28	0.25
HLA-DQA/B	rs1794275	A	0.23	0.20	0.22
This study					
ITGAX/ITGAM	**rs7190997**	**C**	**0.47**	**0.53**	**0.75**
ACCS	**rs2074038**	**T**	**0.00**	**0.12**	**0.33**
ODF1/ KLF10	rs2033562	G	0.67	0.33	0.59
ST6GAL1	**rs7634389**	**C**	**0.16**	**0.38**	**0.40**
DEFA	**rs2738058**	T	**0.35**	**0.40**	**0.65**
DEFA	**rs9314614**	C	**0.19**	**0.41**	**0.43**
DEFA	rs12716641	T	0.76	0.58	0.79

Supplementary table 16. Risk allele frequencies of SNPs in previously reported and newly discovered loci for IgAN in HapMap Africans (YRI), Europeans (CEU) and Asians (CHB+JPT). Frequency information was extracted from HapMap Data Rel 28 Phase II+III and 1000 genomes pilot low coverage sequencing data. Loci showing a trend of increasing risk allele frequencies from Africans to Asians are highlighted in bold font (http://hapmap.ncbi.nlm.nih.gov/)

Supplementary Note. Description of sample collections

GWAS cohorts

The original genome-wide discovery dataset consisted of 1,523 cases recruited from The First Affiliated Hospital, Sun Yat-sen University.

Validation 1 cohorts

This cohort consists of 2,096 individuals of Han Chinese ancestry (2,651 biopsy-diagnosed cases and 2,907controls)recruited across China, including the clinical centers of Guangdong, Guangxi, Sichuan, Henan, Fujian, Hunan, Hubei,Jiangxi, Jiangsu and Yunnan. All cases had a histology-proven diagnosis of IgAN. Genomic DNA was isolated from whole blood using a commercial DNA extraction kit (Qiagen) and quantified using Picogreen reagent (Invitrogen).

- The Guangdong Validation 1 cohorts (1,008 cases and 2,907 controls)was recruited among self-reported Han Chinese individuals residing in the metropolitan areas of five major cities in Guangdong: Guangzhou (The First Affiliated Hospital of Sun Yat-sen University, Guangdong General Hospital, The Third Affiliated Hospital of Sun Yat-sen University, ZhuJiang Hospital of Nanfang Medical University, Guangzhou General Hospital of Nanjing Military Commond, Nanfang Hospital of Nanfang Medical University, The Second Affiliated Hospital of Sun Yat-sen University, Guangdong Provincial Traditional Chinese Medicine Hospital and The Affiliated Hospital of Guangdong Medical University), Foshan (The First People’s Hospital of Foshan), Zhuhai (The Fifth Affiliated Hospital of Sun Yat-sen University), Zhongshan(The People’s Hospital of Zhongshan), and Shenzhen (The First Affiliated Hospital of ShenZhen University).
- The Guangxi Validation 1 cohort (62 cases) was composed of self-reported Han Chinese individuals recruited in Nanning (The First Affiliated Hospital of Guangxi Medical University and The People’s Hospital of Guangxi Zhuang Autonomous Region).
- The Sichuan Validation 1 cohort (119 cases) was composed of self-reported Han Chinese individuals recruited in Chengdu (West China Medical School, West China Hospital, Sichuan University).
- The Henan Validation 1 cohort (2 cases) was composed of self-reported Han Chinese individuals recruited in Zhengzhou (The First Affiliated Hospital of Zhengzhou University).
- The Fujian Validation 1 cohort (265 cases) was recruited among self-reportedHan Chinese individuals residing in the metropolitan areas of two major cities in Fujian: Fuzhou (Fuzhou General Hospital of Nanjing Military Command and The First Affiliated Hospital of Fujian Medical University)and Xiamen(The First Affiliated Hospital of ShenZhen University).
- The Hunan Validation 1 cohort (2 cases) was composed of self-reported Han Chinese individuals recruited in Changsha (The Third Xiangya Hospital of Central South University).
- The Hubei Validation 1 cohort (59 cases) was composed of self-reported Han Chinese individuals recruited in Wuhan (Tongji Hospital, Tongji Medical College, Huazhong University of Science and Technolog, Union Hospital, Tongji Medical College, Huazhong University of Science and Technology and Wuhan First Municipal People’s Hospital).
- The Jiangxi Validation 1 cohort (14 cases) was composed of self-reported Han Chinese individuals recruited in Nanchang (The Jiangxi Provincial People’s Hospital and The First Affiliated Hospital of Nanchang University).
- The Jiangsu Validation 1 cohort (249 cases) was composed of self-reported Han Chinese individuals recruited in Nanjing (Nanjing General Hospital of Nanjing Military Commond).
- The Yunnan Validation 1 cohort (4 cases) was composed of self-reported Han Chinese

individuals recruited in Kunming (YunNan Provincial People's Hospital).

- Unknown Validation 1 cohorts (867 cases)

Validation 2 cohorts

This cohort consists of 6,630 individuals of Han Chinese ancestry (2,428 biopsy-diagnosed cases and 4,202 controls) recruited across China, including the clinical centers of Guangdong, Guangxi, Shanghai, Ningxia, Henan, Zhejiang, Sichuan, Jiangxi, Jiangsu, Fujian, Hubei, Shanxi and Beijing. All cases had a histology-proven diagnosis of IgAN. Genomic DNA was isolated from whole blood using a commercial DNA extraction kit (Qiagen) and quantified using Picogreen reagent (Invitrogen).

- The Guangdong Validation 2 cohort (300 cases and 1,800 controls) was recruited among self-reported Han Chinese individuals residing in the metropolitan areas of three major cities in Guangdong: Guangzhou (The First Affiliated Hospital of Sun Yat-sen University, Guangdong General Hospital, ZhuJiang Hospital of Nanfang Medical University, The First Affiliated Hospital of Jinan University and Guangdong General Hospital), Foshan (The First People's Hospital of Foshan), and Shenzhen (The First Affiliated Hospital of ShenZhen University).
- The Guangxi Validation 2 cohort (114 cases) was composed of self-reported Han Chinese individuals recruited in Nanning (The First Affiliated Hospital of Guangxi Medical University and The People's Hospital of Guangxi Zhuang Autonomous Region).
- The Shanghai Validation 2 cohort (326 cases) was composed of self-reported Han Chinese individuals recruited in Shanghai (RuiJin Hospital Affiliated To Shanghai Jiao Tong University School of Medicine and Shanghai Changzheng hospital, Affiliated To The Second Military Medical University).
- The Ningxia Validation 2 cohort (193 cases) was composed of self-reported Han Chinese individuals recruited in Yinchuan (General Hospital of Ningxia Medicine University).
- The Henan Validation 2 cohort (42 cases) was composed of self-reported Han Chinese individuals recruited in Zhengzhou (The First Affiliated Hospital of Zhengzhou University).
- The Zhejiang Validation 2 cohort (25 cases) was composed of self-reported Han Chinese individuals recruited in Hangzhou (The First Affiliated Hospital of College of Medicine, Zhejiang University).
- The Sichuan Validation 2 cohort (11 cases) was composed of self-reported Han Chinese individuals recruited in Chengdu (West China Medical School, West China Hospital, Sichuan University).
- The Jiangxi Validation 2 cohort (28 cases) was composed of self-reported Han Chinese individuals recruited in Nanchang (The First Affiliated Hospital of Nanchang University).
- The Jiangsu Validation 2 cohort (15 cases) was composed of self-reported Han Chinese individuals recruited in Nanjing (Nanjing General Hospital of Nanjing Military Commond).
- The Fujian Validation 2 cohort (40 cases and 252 controls) was recruited among self-reported Han Chinese individuals residing in Fuzhou (Fuzhou General Hospital of Nanjing Military Commond and The First Affiliated Hospital of Fujian Medical University).
- The Hubei Validation 2 cohort (64 cases) was composed of self-reported Han Chinese individuals recruited in Wuhan (Tongji Hospital, Tongji Medical College, Huazhong University of Science and Technolog, Union Hospital, Tongji Medical College, Huazhong University of Science and Technology).
- The Shanxi Validation 2 cohort (350 controls) was composed of self-reported Han Chinese individuals recruited in Taiyuan (the Second Affiliated Hospital of Shanxi Medical University).
- The Beijing Validation 2 cohort (1,270 cases and 1,800 controls) was composed of self-reported Han Chinese individuals recruited in Beijing (Peking University First Hospital).

Validation 3 cohorts

This cohort consists of 1,800 individuals of Han Chinese ancestry (1800 biopsy-diagnosed cases and 1,910 age/gender/region matched controls) recruited across China, including the clinical centers of Guangdong, Guangxi, Shanghai, Hebei, Sichuan, Jiangxi, Shaanxi, Fujian, Hunan, Hubei, Ningxia, Shanxi, Shandong, Yunnan, Heilongjiang, Henan, Gansu, Liaoning, Hainan and Beijing. All cases had a histology-proven diagnosis of IgAN. Genomic DNA was isolated from whole blood using a commercial DNA extraction kit (Qiagen) and quantified using Picogreen reagent (Invitrogen).

- The Guangdong Validation 3 cohort (380 cases and 1,910 controls) was recruited among self-reported Han Chinese individuals residing in the metropolitan areas of three major cities in Guangdong: Guangzhou (The First Affiliated Hospital of Sun Yat-sen University, The Third Affiliated Hospital of Sun Yat-sen University, ZhuJiang Hospital of Nanfang Medical University, Guangdong General Hospital), Foshan (The First People's Hospital of Foshan), Huizhou(Huizhou Municipal Central Hospital), and Jiangmen (JiangMen Municipal Central Hospital).
- The Guangxi Validation 3 cohort (18 cases) was composed of self-reported Han Chinese individuals recruited in Nanning (The First Affiliated Hospital of Guangxi Medical University and The People's Hospital of Guangxi Zhuang Autonomous Region).
- The Shanghai Validation 3 cohort (195 cases) was composed of self-reported Han Chinese individuals recruited in Shanghai (RuiJin Hospital Affiliated To Shanghai Jiao Tong University School of Medicine, XinHua Hospital Affiliated To Shanghai Jiao Tong University School of Medicine, The Sixth People's Hospital of ShanHai, and ShangHai HuaShan hospital Affiliated To FuDan University).
- The Ningxia Validation 3 cohort (26 cases) was composed of self-reported Han Chinese individuals recruited in Yinchuan (General Hospital Of NingXia Medicine University).
- The Hebei Validation 3 cohort (13 cases) was composed of self-reported Han Chinese individuals recruited in Shijiazhuang (The Third Hospital of Hebei Medical University, HeBei Provincial People's Hospital Hospital).
- The Sichuan Validation 3 cohort (474 cases) was composed of self-reported Han Chinese individuals recruited in Chengdu (Sichuan Provincial People's Hospital). ChongQing (The First Affiliated Hospital of ChongQing Medical University, Xinqiao hospital Affiliated To Third Military Medical University).
- The Jiangxi Validation 3 cohort (33 cases) was composed of self-reported Han Chinese individuals recruited in Nanchang (The First Affiliated Hospital of Nanchang University and The Second Affiliated Hospital of Nanchang University).
- The Shaanxi Validation 3 cohort (10 cases) was composed of self-reported Han Chinese individuals recruited in Xian (XiJing Hospital, Affiliated To The Fourth Military Medical University).
- The Fujian Validation 3 cohort (15 cases) was recruited among self-reported Han Chinese individuals residing in Fuzhou (Fuzhou General Hospital of Nanjing Military Commond and The First Affiliated Hospital of Fujian Medical University).
- The Hubei Validation 3 cohort (3 cases) was composed of self-reported Han Chinese individuals recruited in Wuhan (Tongji Hospital, Tongji Medical College, Huazhong University of Science and Technolog, and ZhongNan Hospital of WuHan University).
- The Hunan Validation 3 cohort (32 cases) was composed of self-reported Han Chinese individuals recruited in Wuhan (The Second Xiangya Hospital of Central South University and The Third Xiangya Hospital of Central South University).
- The Shanxi Validation 3 cohort (4 cases) was composed of self-reported Han Chinese individuals recruited in Taiyuan (ShanXi Provincial People's Hospital).

- The Beijing Validation 3 cohort (511 cases) was composed of self-reported Han Chinese individuals recruited in Beijing (Peking University First Hospital, Beijing Anzhen Hospital Affiliated To Capital Medical University, Beijing XuanWu hospital Affiliated To Capital Medical University).
- The Shandong Validation 3 cohort (15 cases) was composed of self-reported Han Chinese individuals recruited in Jinan (Shandong Provincial Hospital) and Qingdao(The Affiliated Hospital of QingDao University).
- The Hainan Validation 3 cohort (2 cases) was composed of self-reported Han Chinese individuals recruited in HaiKou (The People's Hospital of HaiKou).
- The Liaoning Validation 3 cohort (23 cases) was composed of self-reported Han Chinese individuals recruited in Dalian(The First Affiliated Hospital of DaLian Medical University) and Shenyang(The First Affiliated Hospital of China Medical University).
- The Gansu Validation 3 cohort (7 cases) was composed of self-reported Han Chinese individuals recruited in Lanzhou (LanZhou General Hospital of LanZhou Military Commond, The Second Affiliated Hospital of LanZhou University).
- The Henan Validation 3 cohort (15 cases) was composed of self-reported Han Chinese individuals recruited in Zhengzhou (The First Affiliated Hospital of Zhengzhou University).
- The Heilongjiang Validation 3 cohort (2 cases) was composed of self-reported Han Chinese individuals recruited in HarBin (The Second Affiliated Hospital of HarBin Medical University).
- The Yunnan Validation 3 cohort (22 cases) was composed of self-reported Han Chinese individuals recruited in Kunming (First People's Hospital of Yunnan Province).

Classification of Northern and Southern Chinese subjects

The definition of northern and southern was done in the same way as our previous GWAS study[2]. Briefly, subjects originating from (Hai Nan, Guang Dong, Guang Xi, Fu Jian, Yu Nan, Gui Zhou, Hu Nan, Hubei, Si Chuan, Jiang Xi, Jiang Shu, Zhe Jiang, Shang Hai, An Hui and Chong Qing provinces) of China were classified as southern Chinese and the remaining subjects were classified as northern Chinese.

Grading of samples by the Oxford classification[17,18]

A total of 2,776 IgAN patients from our centre (1,248 from the discovery samples and 1,528 from the validation samples) had full pathological information graded by the Oxford classification. The number and percentage of samples graded by Oxford classification are as shown below:

Number and percentage of samples graded by Oxford classification			
M: n=2024	MO:821(40.6%)	M1:1203(59.4%)	
E: n=1892	E0:1559(82.4%)	E1:333(17.6%)	
S: n=2035	S0:1043(51.3%)	S1: 992(48.7%)	
T: n=1949	T0:1301(66.8%)	T1:494(25.3%)	T2:154(7.9%)

Supplementary references

1. Zhou, X. & Stephens, M. Genome-wide efficient mixed-model analysis for association studies. Nat Genet 44, 821-4 (2012).
2. Yu, X.Q. et al. A genome-wide association study in Han Chinese identifies multiple susceptibility loci for IgA nephropathy. Nat Genet 44, 178-82 (2012).
3. Purcell, S., Cherny, S.S. & Sham, P.C. Genetic Power Calculator: design of linkage and association genetic mapping studies of complex traits. Bioinformatics 19, 149-50 (2003).
4. Gharavi, A.G. et al. Genome-wide association study identifies susceptibility loci for IgA nephropathy. Nat Genet 43, 321-7 (2011).
5. Kiryluk, K. et al. Discovery of new risk loci for IgA nephropathy implicates genes involved in immunity against intestinal pathogens. Nat Genet (2014).
6. Jia, X. et al. Imputing amino acid polymorphisms in human leukocyte antigens. PLoS One 8, e64683 (2013).
7. Westra, H.J. et al. Systematic identification of transeQTLs as putative drivers of known disease associations. Nat Genet 45, 1238-43 (2013).
8. Fairfax, B.P. et al. Genetics of gene expression in primary immune cells identifies cell type-specific master regulators and roles of HLA alleles. Nat Genet 44, 502-10 (2012).
9. Boyle, A.P., et al. Annotation of functional variation in personal genomes using RegulomeDB. Genome Research 22, 1790-1797 (2012)
10. Ward, L.D. & Kellis, M. HaploReg: a resource for exploring chromatin states, conservation, and regulatory motif alterations within sets of genetically linked variants. Nucleic Acids Res 40, D930-4 (2012).
11. Welter, D. et. al.. The NHGRI GWAS Catalog, a curated resource of SNP-trait associations. *Nucleic Acids Research* 42 (Database issue), D1001-D1006 (2014)
12. Kunisawa, J. *et al.* Microbe-dependent CD11b+ IgA+ plasma cells mediate robust early-phase intestinal IgA responses in mice. *Nat. Commun.* **4**, 1772 (2013).
13. Hom,G. et. al. Association of systemic lupus erythematosus with C8orf13-BLK and ITGAM-ITGAX. N Engl J Med 358,900-9 (2008)
14. Harley, J.B. et al. Genome-wide association scan in women with systemic lupus erythematosus identifies susceptibility variants in ITGAM, PXK, KIAA1542 and other loci. Nat Genet40, 204-10 (2008)
15. Zhou, X.J. et al. Association of systemic lupus erythematosus susceptibility genes with IgA nephropathy in a Chinese cohort. Clin J Am Soc Nephrol 9, 788-97 (2014).
16. Corrado, A., Quarta, L., Di Palma, A.M., Gesualdo, L. &Cantatore, F.P. IgA nephropathy in systemic lupus erythematosus. Clin Exp Rheumatol 25, 467-9 (2007).
17. Cattran D.C. et al. The Oxford classification of IgA nephropathy: rationale, clinicopathological correlations, and classification. Kidney Int 76, 534-45. (2009)
18. Roberts I.S. et al. The Oxford classification of IgA nephropathy: pathology definitions, correlations, and reproducibility. Kidney Int 76, 546-56. (2009)
